# Llama Nanoantibodies with Therapeutic Potential against Human Norovirus Diarrhea

**DOI:** 10.1371/journal.pone.0133665

**Published:** 2015-08-12

**Authors:** Lorena Garaicoechea, Andrea Aguilar, Gabriel I. Parra, Marina Bok, Stanislav V. Sosnovtsev, Gabriela Canziani, Kim Y. Green, Karin Bok, Viviana Parreño

**Affiliations:** 1 Instituto de Virología, INTA, Castelar, Buenos Aires, Argentina; 2 Laboratory of Infectious Diseases, NIAID, National Institutes of Health, Bethesda, Maryland, United States of America; 3 Instituto Leloir, Buenos Aires, Argentina; Tulane University, UNITED STATES

## Abstract

Noroviruses are a major cause of acute gastroenteritis, but no vaccines or therapeutic drugs are available. Llama-derived single chain antibody fragments (also called VHH) are small, recombinant monoclonal antibodies of 15 kDa with several advantages over conventional antibodies. The aim of this study was to generate recombinant monoclonal VHH specific for the two major norovirus (NoV) genogroups (GI and GII) in order to investigate their potential as immunotherapy for the treatment of NoV diarrhea. To accomplish this objective, two llamas were immunized with either GI.1 (Norwalk-1968) or GII.4 (MD2004) VLPs. After immunization, peripheral blood lymphocytes were collected and used to generate two VHH libraries. Using phage display technology, 10 VHH clones specific for GI.1, and 8 specific for GII.4 were selected for further characterization. All VHH recognized conformational epitopes in the P domain of the immunizing VP1 capsid protein, with the exception of one GII.4 VHH that recognized a linear P domain epitope. The GI.1 VHHs were highly specific for the immunizing GI.1 genotype, with only one VHH cross-reacting with GI.3 genotype. The GII.4 VHHs reacted with the immunizing GII.4 strain and showed a varying reactivity profile among different GII genotypes. One VHH specific for GI.1 and three specific for GII.4 could block the binding of homologous VLPs to synthetic HBGA carbohydrates, saliva, and pig gastric mucin, and in addition, could inhibit the hemagglutination of red blood cells by homologous VLPs. The ability of Nov-specific VHHs to perform well in these surrogate neutralization assays supports their further development as immunotherapy for NoV treatment and immunoprophylaxis.

## Introduction

Noroviruses (NoV), members of the family *Caliciviridae*, are the major cause of epidemic gastroenteritis [1,2]. A gastroenteritis episode due to NoV is incapacitating during the acute phase that usually lasts from 1 to 3 days and can include explosive vomiting, stomach cramps, and diarrhea. Immunocompetent patients characteristically recover quickly, but the illness can become life-threatening in young, elderly and immunocompromised populations [3–9]. Virus shedding in stool can be detected for approximately 30 days in healthy individuals [10], but in immunocompromised patients, virus shedding can persist for months to years [2,11,12]. It has been proposed that long-term virus shedding may contribute to the spread of the virus [13–15].

The single-stranded positive-sense RNA genome is surrounded by a non-enveloped capsid formed by the major capsid protein VP1 and a minor structural protein VP2 [1]. Crystallographic analysis showed that the NoV capsid is formed from 180 molecules of VP1, organized into 90 dimers [16]. Each VP1 monomer is divided into two domains designated shell (S) and protruding (P), linked by a flexible hinge [16].

Noroviruses are divided into six major genogroups designated Genogroup (G) I through GVI. Genogroups I and II contain the majority of NoV strains associated with human disease and are further subdivided into 9 and 21 genotypes, respectively [17]. Although NoV GI.1 was the first genotype described, the GII.4 genotype has been associated with the majority of global outbreaks since the mid-1990s, when active surveillance with molecular diagnostic techniques was initiated [5,18–23]. Norovirus vaccines comprised of recombinant virus-like particles (VLPs) [24] are currently under evaluation but the number of antigenic components needed for broad protection is not known [25–27]. The local immune response was genotype-specific in our recent study of natural infection and failed to protect against subsequent illness with a different genotype [28], consistent with other studies [10] and a need for multivalent vaccines.

Crystallographic studies showed that NoV bind carbohydrates of the human histo–blood group antigens (HBGAs) through the protruding domain of VP1 [29,30]. It has been proposed that this binding facilitates viral entry into the epithelial cells of the gastrointestinal tract. [31] Susceptibility to NoV in humans has been linked to an allelic variation of HBGA secretor status, where expression of HBGA antigens on epithelial surfaces (in secretor positive individuals) provides attachment ligands for the virus in the gut [32]. Thus, the measurement of antibodies that block the interaction between VLPs and HBGA carbohydrates has been developed as a surrogate assay for virus neutralization. Several sources of carbohydrates have been used in these surrogate neutralization tests that include saliva, pig gastric mucin (PGM) and synthetic carbohydrates, and [33,34]. For the surrogate neutralization assay using synthetic HBGA a correlation of antibody titers with protection to NoV infection has been established [35]. A hemagglutination inhibition (HAI) assay using human red blood cells (RBC) has also been used as a surrogate neutralization test because a correlation was found between HAI serum antibody titers and susceptibility to infection in volunteer challenge studies [36,37].

Murine-origin conventional monoclonal antibodies (MAbs) have been shown to block the interaction of VLPs with intact cells or synthetic HBGA molecules [22,38], and five HBGA-blocking sites have been characterized recently [14,20,23,39,40]. Additional approaches to the generation of NoV-specific monoclonal antibodies include the isolation of single chain antibodies (scFv) from primed humans and chimpanzees that demonstrate activity in surrogate neutralization assays [41,42]. The latter antibodies are attractive for immunotherapy, as they would not require humanization.

An emerging alternative approach to immunotherapy is the technology of llama-derived VHH recombinant monoclonal antibodies, also known as “nanobodies” [43–45]. The IgG_2_ and IgG_3_ subtypes of llamas are considered heavy chain antibodies because they lack a light chain; the variable domain of these antibodies is called VHH and is comprised of only one polypeptide chain. The VHH domain is approximately 15 kDa and is the smallest known antigen recognition site that occurs in mammals, with full binding capacity and affinities comparable to conventional antibodies [43]. Through the generation of cDNA libraries from the immune cells of immunized llamas, VHH antibodies specific for a targeted antigen can be developed as recombinant monoclonal proteins [43,45]. As they are comprised of only one polypeptide chain, VHH antibodies are amenable to expression in a number of protein production platforms [43,45–49]. They are highly resistant to protease activity and non-immunogenic when administered by the oral route, a strong advantage for consideration in the development of local treatment for enteric pathogens [44–47]. Furthermore, they are non-immunogenic when administered by the intravenous route and can be engineered into any human immunoglobulin scaffold, providing an approach for systemic therapies [50–53].

This work reports the development of recombinant monoclonal VHH specific for two distinct human NoV genogroups, GI.1 (Norwalk) and GII.4 (MD2004) and their performance in a number of applications including HBGA blocking assays. The results indicate that these new antibodies hold considerable promise as immunological tools for norovirus research and therapeutic development.

## Materials and Methods

### Expression and purification of VLPs

Norovirus VLPs containing VP1 and VP2 capsid proteins were expressed in a baculovirus system as described previously [21,28,54–58].

For the different assays of this work VLPs of the following NoV strains were included: Hu/NoV/GI.1/Norwalk/1968/U.S., Hu/NoV/GI.1/P7-587/2007/Stromstad/SE; Hu/NoV/GI.3/Desert-Shield395/1990/U.S.; Hu/NoV/GII.4/MD2004/2004/U.S., Hu/NoV/GII.4/MD145-12/1987/U.S.; Hu/NoV/GII.1/Hawaii/1971/U.S; Hu/NoV/GII.3/Toronto24/1991/CA; Hu/NoV/GII.4/CHDC4871/1977/U.S.; Hu/NoV/GII.4/HS-191/2001/U.S., Hu/NoV/GII.3/Aus2001/2001/AU, Hu/NoV/GII.3/Aus2007/2007/AU, Hu/NoV/GII.3/Aus2008/2008/AU, Hu/NoV/GII.3/CHDC2005/2005/U.S., Hu/NoV/GII.3/CHDC5261/1990/U.S., Hu/NoV/GII.3/CHDC4031/1988/U.S.; Hu/NoV/GII.3/CHDC32/1976/U.S., Hu/NoV/GII.7/DC119/1978/U.S., Hu/NoV/GII.3/Maizuru/2000/JP, Hu/NoV/GII.14/M7/1999/U.S., Hu/NoV/GI.5/SzUG1 /1997-99/JP, Hu/NoV/GI.6/Hesse /1997/GE, Hu/NoV /GII.2/ Snow Mountain/1976/U.S., Hu/NoV/GII.4/RockvilleD1/2012/U.S., and Hu/NoV/GII.6/Bethesda/2012/U.S. As negative controls, VLPs derived from Hu/NoV/GIV.1/St.Cloud 624/1998/U.S., Stellar sea lion vesivirus V1415 and Mink calicivirus were included.

For determination of the specificity of the VHH against the different subdomains of VP1, the VLP NV S domain/MD2004 P domain chimera and the VLP MD2004 S domain/NV P domain chimera were utilized as previously described [40]. Mutagenized VLPs designated MD2004 G340A/E376Q and MD2004 AD294GI [40] were used in epitope mapping of GII.4 VHH M7.

### Llama immunization

Two male llamas of one year of age were immunized by intramuscular injection with 3 doses (day 0, 30 and 73) or 4 doses (day 0, 30, 73 and 225) of immunogen containing approximately 300 μg/per dose of NoV VLP from the Norwalk virus strain (Hu/NoV/GI.1/Norwalk/1968/U.S.), or the MD2004 strain (Hu/NoV/GII.4/MD2004/ 2004/U.S.), respectively. For the first immunization, VLPs were emulsified in complete Freund´s adjuvant. The following immunizations were formulated in incomplete Freund´s adjuvant. Serum and blood samples were taken at days 0, 4 and 7 after each inoculation. The antibody responses to NoV in serum during the time course of immunization were monitored by ELISA. To evaluate the effector B-cell response, an ELISPOT assay determining the number of NoV-specific antibody-secreting cells was performed at 4 and 7 days post each inoculation. Llama management, inoculation, and sample collection were conducted by trained personnel under the supervision of a DVM, PhD Gisela Marcoppido as well as a technician specialized in llama welfare, in accordance with Argentinean and international guidelines of animal welfare. This study was approved by the Animal Care and Use Committee of INTA (CICUAE) under the protocol N° 08/2011. The llamas were housed in a paddock especially prepared for the VHH line of research. The paddock is located at the INTA Castelar Campus and encompasses a total space of 20 meters by 40 meters. The animals had a roof as well as a separate area where experimental procedures such as vaccination and sample collection were conducted. The animals also had access to food and water at all times, 24 hours a day, 7 days a week. Additionally, the llamas were free to roam in open space during the day, but were kept in a smaller roofed area at night. After the experiment was completed, the llamas stayed in the paddock for six months in order to confirm the absence of adverse effects due to the immunization and blood sampling. Since the llamas did not receive any infectious agents, they subsequently were sent back to their farm of origin.For the ELISA or ELISPOT assays 96 flat bottom well Maxisorp ELISA plates (NUNC, Thermo Scientific, U.S.) were coated with 50 ng of NoV VLPs diluted in 50 μl of PBS pH 7.4 ON at 4°C (Norwalk or MD2004 VLPs were used). The plates were washed with 0.05% Tween 20-PBS, and then were blocked with 200 μl of 5% skim milk in PBS pH 7.4 for 1 h at 37°C and then washed with 0.05% Tween 20-PBS. For IgG detection by ELISA, 50 μl of each serum sample was added per well (in duplicate) beginning with a dilution of 1:50 in 1% skim milk-PBS, serial four-fold dilutions were made in the plate and then the plates were incubated at 37°C for 2 h. For Antibody Secreting Cell detection by ELISPOT, suspensions of MNC derived from peripheral blood of the inoculated llamas were added to wells (in quadruplicate) and subjected to serial ten-fold dilutions, starting with 1x10^6^ cells/well. After centrifugation at 500 g for 5 min, plates were incubated for 12 to 14 h at 37°C in 5% CO_2_ atmosphere. After the incubation period, the plates were washed with 0.05% Tween 20-PBS and a peroxidase-labeled anti-Llama IgG (Bethyl Labs, U.S.) at a 1:1,500 dilution in 1% skim milk-PBS was added at 50 μl /well. Following incubation at 37°C for 1 h for ELISA or 2 h for ELISPOT, the plates were washed with 0.05% Tween 20-PBS. The ELISA assay was developed with commercial ABTS (2,2azinobis(3-ethylbenzthiazolinesulfonicacid)) (KPL, U.S.) /H_2_O_2_ substrate, added at 100 μl/well. The absorbance at 405nm was read in an ELISA reader (Multiskan EX, Thermo scientific) and averages of duplicate wells were used in the calculation of the values. Samples were designated positive when the measured absorbance at Abs405nm exceeded absorbance of the control wells plus 3 SD. The ELISPOT assay was developed with 50 μl of a tetramethylbenzidine (TMB) peroxidase substrate system (KPL, U.S.) and the spots counted under the microscope.

### VHH library production

Two VHH libraries were developed from circulating lymphocytes of the two immunized llamas. From the llama immunized with VLPs of NoV MD2004 (GII.4) 300 ml of blood were taken 4 days after the third dose and 300 ml of blood 4 days after the fourth dose. From the other llama immunized with VLPs of NoV Norwalk (GI.1), 450 ml of blood were taken 4 days after the third dose. Mononuclear cells were isolated by Ficoll-Paque Plus (GE Healthcare, U.S.) gradient centrifugation, pelleted, frozen in liquid nitrogen, and stored at -80°C until used. The total RNA was extracted with a commercial RNA extraction kit, Nucleospin RNA midi (Macherey Nagel, Germany), yielding 300 μg of RNA for the Norwalk- and 420μg of RNA for the MD2004-immunized llamas. Subsequently, first-strand cDNA was synthesized from the total RNA by using MMLV Reverse Transcriptase (Promega, U.S.), with random primers (Life Technologies, U.S.) according to the manufacturer instructions. For each 20 μl reaction, 10 μg of total RNA were added. The VHH-repertoire was PCR amplified from the total RNA for each llama as described previously [59].

PCR amplification products were purified, digested with SfiI and NotI restriction enzymes, and cloned into the corresponding sites of the phagemid vector pAO-Lib [60]. Ligated material was transformed into *Escherichia coli* TG1 cells by electroporation. Colonies were harvested by scraping in culture medium, washed and stored at -80°C in LB medium supplemented with glycerol (30% final concentration).

### Enrichment in VHH of interest

The bacteria containing the VHH library were infected with M13K07 helper phages (Life Technologies, U.S.), and phage particles expressing the VHH repertoire were rescued and precipitated with polyethylene glycol as described previously [61]. Enrichment for specific binders was performed with the phage display technology through two rounds of *in vitro* selection (biopanning). Briefly, 96 flat bottom well Maxisorp ELISA plates (NUNC Thermo Scientific, U.S.) were coated with 100μl/well of NoV VLPs from Norwalk or from MD2004 strains (100 ng/well in carbonate buffer pH 9.6) ON at 4°C. The plates were washed with 0.05% Tween 20-PBS, and then were blocked with 200 μl of 5% skim milk in PBS pH 7.4 for 1 h at 37°C. After another wash with 0.05% Tween 20-PBS phages from each library were added to the plates according to the different biopanning strategies and incubated for 1 h at room temperature.

Two strategies of selection were performed, one in which phages were incubated directly with the homologous VLPs, and the other in which phages were additionally pre-incubated with heterologous VLPs (of different Genogroup) to subtract cross-reactive phages and enhance specificity. After incubation, the plates were washed with 0.05% Tween 20-PBS and bound phage particles were eluted with 100 mM triethylamine (pH 10.0) and immediately neutralized with 1M Tris (pH 7.4). The eluted phages were used to infect exponentially growing TG1 cells. After the second round of biopanning, individual colonies from the NV and MD2004 NoV libraries (200 colonies each), were grown, and the corresponding VHH clones were analyzed by phage ELISA for specificity to NoV GI.1 (Norwalk) and NoV GII.4 (MD2004).

### Screening for GI.1 and GII.4 specific VHH fragments by phage ELISA

Phages displaying the selected VHH were produced by the individual TG1 *Escherichia coli* clones as previously described [62]. Ninety-six flat bottom well Maxisorp ELISA plates (NUNC Thermo Scientific, U.S.) were coated with 100 μl/well of NoV VLPs from Norwalk or from MD2004 strains (100 ng/well in carbonate buffer pH 9.6) ON at 4°C). and then were blocked with 5% skim milk in PBS pH 7.4 for 1 h at 37°C. Then phages from each clone were added to wells coated with the different NoV VLPs and blank coated wells. The assay was developed with a monoclonal antibody anti-M13 p8 (GE Healthcare, U.S.) at a 1/5,000 dilution in 1% skim milk-PBS at 50 μl /well and then an anti-mouse IgG conjugated with peroxidase (KPL, U.S.) at a 1/2,000 dilution. Finally, commercial ABTS (KPL, U.S.)/H_2_O_2_ substrate, was added at 100μl/well. The absorbance at 405 nm was read in an ELISA reader (Multiskan EX, Thermo scientific).

### Expression and purification of recombinant VHH

VHH cDNA of 11 clones that scored positive in phage ELISA for NoV VLPs of Norwalk strain (GI.1) or MD2004 strain (GII.4) were subcloned using the restriction enzymes SfiI and NotI into the expression vector pHEN6 [62]. Expression of recombinant monovalent VHH was performed as previously described [45]After the expression the bacteria were pelleted, the periplasmic proteins were extracted by osmotic shock [63] and the VHH were purified from this periplasmic extract by using a High-Trap HP Ni-chelating column (GE Healthcare, U.S.).

The VHH nucleotide sequences of the obtained VHH clones were aligned by ClustalW with Mega 6.06 and the alignment was edited with BioEdit.

### Specificity of VHH in Norovirus VLP ELISA

Briefly, 96 U bottom well vinyl microtiter plates (Thermo Scientific, U.S.), were coated with 100 ng of purified VLPs/well diluted in 50 μl of PBS pH 7.4 ON at 4°C. Wells coated with PBS alone were used as a negative control for VHH binding. The plates were washed with 0.1% Tween 20-PBS, and then were blocked with 200 μl of 5% skim milk in PBS pH 7.4 for 1 h at 37°C. After washing with 0.05% Tween 20-PBS, 50 μl/well of the corresponding VHH dilution (two fold dilutions ranging from 16 to 0.05 ng/well for cuantitative assays and 20 ng/well for cualitative assays) in 5% skim milk in PBS pH 7.4 was added and the plates were incubated for 2 h at room temperature. The plates were washed with 0.05% Tween 20-PBS and a rabbit anti-VHH serum at a 1:8,000 dilution in 5% skim milk in PBS was added at 50 μl /well. Following incubation at 37°C for 1 h, the plates were washed with 0.05% Tween 20-PBS and the binding of antibodies to the VLP antigen was detected with 50 μl /well of a goat anti-rabbit IgG horseradish peroxidase (HRP)–conjugated (KPL, U.S.) at a 1:2,000 dilution in 1% skim milk in PBS. After 1 h of incubation at 37°C, the plates were washed with 0.05% Tween 20-PBS and the assay was developed with commercial ABTS (KPL U.S.)/H_2_O_2_ substrate, added at 100 μl/well._._ The absorbance at 405nm was read in an ELISA reader (Multiskan EX, Thermo scientific). The cut off was defined as twice the absorbance obtained in the blank wells. For determination of the VHH detection limit for GI.1 (Norwalk and P7-587) and GII.4 (MD2004 and MD145) VLPs, serial ten-fold dilutions of VHH were tested from 500 ng/well to 0.005 ng/well against the corresponding VLP. To test the ability of each VHH to recognize VLPs from different NoV strains, a fixed amount of 20 ng of VHH/well was selected.

To evaluate the specificity of the VHH against different domains of VP1, chimeric VLPs Norwalk S/MD2004 P and MD2004 S/Norwalk P were utilized as previously described [40]. The VHH were tested against each chimeric VLP at a fixed concentration of 20 ng per well.

### Western blot analysis

The reactivity of each VHH was analyzed by Western blot (WB). For this assay, 1.5 μg of Norwalk or MD2004 VLPs were mixed with NOVEX 2X Tris-Glycine SDS loading buffer (Life Technologies, U.S.), and after boiling 5 min at 95°C, the samples were subjected to Sodium dodecyl sulfate-polyacrylamide gel electrophoresis (SDS-PAGE) in a NuPAGE Novex 4–12% gel (Life Technologies, U.S.). The proteins were blotted onto a nitrocellulose membrane (Life Technologies, U.S.) using the iBlot Dry Blotting System (Life Technologies, U.S.). The membranes were blocked with 5% skim milk in PBS for 2 h at room temperature. Each VHH (5 μg/ml) was incubated with the transferred VP1 proteins ON at 4°C and the binding was detected by incubating for 1 h at room temperature with rabbit anti-VHH serum at a 1:1,000 dilution. After washing with 0.05% Tween 20-PBS, alkaline Phosphatase–conjugated goat anti-rabbit IgG was added at a 1:2,000 dilution and incubated for 1 h at room temperature. The binding of the conjugate was detected with the NBT-BCIP Chromogenic system (Sigma Aldrich, U.S.).

### HBGA blocking assays

Ninety six flat bottom well Neutravidin coated plates (Pierce Thermo Scientific, U.S.) were used following the manufacturer´s instructions. Briefly, plates were incubated with 1μg/well of biotinylated synthetic carbohydrates H1 (for Norwalk) or H3 (for MD145) (GlycoTech Corporation, U.S.) 1 h at room temperature. The plates were washed with 0.05% Tween 20-PBS. Separately, a total of 150 ng of VLPs diluted in 100 μl of buffer were pre-incubated with 400, 200, 100, 50, 25 and 0 ng of VHH for 1 hour at room temperature. The 150 ng of the pre-incubated VLPs were added to each well of the carbohydrate-coated plates and incubated for 1 h at room temperature. The plates were washed and the binding of captured VLPs was determined with guinea pig hyperimmune serum used at a 1:10,000 dilution and incubated for 1 h at room temperature. After another wash, the plates were incubated with HRP-conjugated goat anti-guinea pig IgG at a 1:2,000 dilution (KPL, U.S.) for 1 h at room temperature. Following a last wash, the assay was developed with commercial ABTS (KPL, U.S.)/H_2_O_2_ substrate, added at 100 μl/well. The absorbance at 405 nm was read in an ELISA reader (Multiskan EX, Thermo scientific). The percent control binding was defined as the binding level in the presence of antibody pretreatment divided by the binding level in the absence of antibody pretreatment multiplied by 100. Mean percentage control binding represents the results of two replicates for each dilution of VHH tested. An antibody was designated as a ‘‘blockade” antibody for a VLP if at least 50% of control binding (EC_50_) was inhibited by 2 μg/ml antibody or less. Blockade data were fitted and EC_50_ values calculated using sigmoidal dose response analysis of non-linear data in GraphPad Prism 5 (available on the internet, graphpad.com). As positive controls for the blocking of the binding of the VLPs, the monoclonal antibody D8 anti-Norwalk [41] and the monoclonal antibody C9 anti-MD145 were included.

Pig gastric mucin Type III (PGM) (Sigma Aldrich, U.S.) (with HBGA type A, Ley and H2) was used as a second NoV VLP antibody-blocking assay as previously described [13]. Briefly, PGM was resuspended in PBS at 5 mg/ml and coated onto 96 U bottom well vinyl microtiter plates (Thermo Scientific, U.S.) at 10 μg/ml in PBS and 100 μl/well for 4 hours at room temperature. Plates were then blocked ON at 4°C in 5% skim milk in 0.05% Tween 20-PBS. Separately, Norwalk and MD2004 VLPs (0.5 mg/ml) were pre-treated with decreasing concentrations of each VHH (2-fold dilutions from 8 μg/ml to 0.125 μg/ml) for 1 hour at room temperature. One hundred μl of the VLPs-VHH mixture were transferred to the PGM coated plates and incubated for 1 h at 37°C. Plates were washed with 0.05% Tween 20-PBS and bound VLPs were detected using specific hyperimmune serum raised in guinea pigs at a 1:10,000 dilution incubated for 1 h at 37°C. After washing, the plates were incubated with an anti-guinea pig IgG-HRP conjugated (KPL, U.S.) at a 1:2,000 dilution for 1h at 37°C. Following a final wash, the assay was developed with commercial ABTS (KPL, U.S.)/H_2_O_2_ substrate, added at 100μl/well. The absorbance at 405nm was read in an ELISA reader (Multiskan EX, Thermo scientific). Mean percentage control binding, EC_50_ and criteria for determination of a blockade VHH were calculated as described above.

A third blocking assay employed saliva as a natural source of HBGA molecules. Saliva positive for Ley antigen from a secretor individual was boiled (95 ˚C) for 10 minutes immediately after collection and centrifuged for 5 min at 13000 g. The pellet was discarded and the clarified saliva supernatant was collected and stored at -20˚C. Ninety six U bottom well vinyl microtiter plates (Thermo Scientific, U.S.) were coated with 100 μl/well of the clarified saliva at a 1:400 dilution in 50 μM carbonate-bicarbonate buffer pH 9.6 and incubated ON at 37 ˚C in a wet atmosphere. In parallel, serial two-fold dilutions of VHH starting from 8 μg/ml were mixed with 1.5 μg/ml of Norwalk or MD145 VLPs and incubated for 1h at 37μC. After 6 washes and a blocking step with 5% skim milk in 0.05% Tween 20-PBS, 50 μl of each VHH-VLP mixture was transferred to the reaction plate, in duplicate. The presence of norovirus VLP was detected with a specific polyclonal guinea pig antiserum raised against the corresponding VLP in guinea pigs at a 1:10,000 dilution incubated for 1h at 37°C. After washing, the plates were incubated with an anti-guinea pig IgG-HRP conjugated (KPL, U.S.) at a 1:2,000 dilution for 1h at 37°C. Following a last wash, the assay was developed with commercial ABTS (KPL, U.S.)/H_2_O_2_ substrate, added at 100μl/well. The absorbance at 405 nm was read in an ELISA reader (Multiskan EX, Thermo scientific). Mean percentage control binding, EC_50_ and criteria for determination of blockade activity were calculated as described above.

### Hemagglutination inhibition assay (HAI)

The hemagglutination inhibition assay (HAI) was performed with human red blood cells (RBC) that were matched for hemagglutination activity with the tested VLPs: MD2004, MD145, NV and P7-587. Blood type 0 Rh- RBCs were selected to perform the assay with GI.1 VLPs (NV and P7-587) and type B Rh+ RBCs were used with GII.4 VLPs (MD2004 and MD145). The samples, RBCs, and buffers were prepared as described elsewhere [36]. The working suspension of RBCs was made by adding 0.75 ml of the RBC pellet to 100 ml of physiologic saline solution, pH 6.2. The VHH samples were diluted in PBS-physiologic saline solution pH 5.5 from 6.25 μg/25 μl to 0.0025 μg/25 μl in two-fold serial dilutions and 25 μl of each dilution was added to each well (in duplicate). Four to eight hemagglutination units (HU) of VLPs were added to each sample dilution and the plates were incubated for 1 h at room temperature. Finally, 50 μl of the RBC working suspension were added to each well and the plates were incubated for 2 h at 4°C. The presence or absence of hemagglutination was observed and the HAI titer of each VHH was defined as the lowest antibody concentration that completely prevented hemagglutination.

### ELISA competition

Briefly, 96 U bottom well vinyl microtiter plates (Thermo Scientific, U.S.) were coated with 12.5 ng/well of VLPs (from Norwalk or MD2004 NoV strains) and incubated ON at 4°C. Plates were washed with 0.05% Tween 20-PBS and blocked with 5% skim milk in PBS for 1 h at room temperature. Then, llama hyperimmune serum specific for the corresponding VLP was added to each well at a 1:500 dilution and followed by incubation for 1 hour at room temperature. After washing the plates, two fold dilutions of each VHH, from 6.25 to 0.012 ng/well were added to the wells in duplicate. A control was included for each VHH in which hyperimmune llama serum was not added. The plates were washed and incubated with anti His MAb (Qiagen, Germany) at a concentration of 10 ng/well for 1 h at room temperature to detect the His-tagged VHH. After washing, incubation with HRP-conjugated anti-mouse immunoglobulin G (KPL, U.S.) at a 1:2,000 dilution for 1 h was performed at room temperature. Following a final wash, the assay was developed with commercial ABTS (KPL, U.S.)/H_2_O_2_ substrate, added at 100 μl/well. The absorbance at 405 nm was read in an ELISA reader (Multiskan EX, Thermo scientific).

In a second assay, each VHH or a pool of all VHHs at a concentration of 250 μg/ml was added to the wells and incubated for 1 h at room temperature. A control for each assay included wells in which blocking VHH was not present. After washing the plates, two fold serial dilutions of the corresponding llama hyperimmune serum from 1:1,000 to 1: 2,000,000 were added to duplicate wells. The assay was developed with HRP-conjugated anti-llama IgG (Bethyl, U.S.) at a 1:2,000 dilution for 1h at room temperature. In this concentration the anti-llama IgG antibody is unable to detect the recombinant VHH. The assay was developed with commercial ABTS (KPL, U.S.)/H_2_O_2_ substrate, added at 100 μl/well. The absorbance at 405 nm was read in an ELISA reader (Multiskan EX, Thermo scientific). The percentage blocking was calculated by comparing the average value in duplicate wells obtained from VLPs pre-incubated with VHH or sera with the average value in wells with VLPs without pre-incubation with VHH or sera. A blocking value of ≤ 50% of binding was considered the cut-off value.

### Immunofluorescence assay competition

Vero cells were plated in 96-well tissue culture plates at 50,000 cells/well and incubated for 24 h at 37°C, 5% CO_2_. The cells were then infected with a modified vaccinia virus expressing bacteriophage T7 RNA polymerase (MVA-T7) at a multiplicity of infection (MOI) of 5 PFU/cell for 1 h at 37°C, 5% CO_2_. After infection, cells were transfected with 400 ng/well of pCI-based plasmid DNA expressing Norwalk or MD2004 VP1 using Lipofectamine 2000 (Life Technologies, U.S.) as previously described [40]. Transfected cells were incubated for 24 h and then fixed with cold methanol for 10 min. The plates were blocked with 10% normal rabbit serum (KPL, U.S.) ON at 4°C. Fifty microliters containing 10 μg/well of each unlabeled VHH and 1 μl of Alexa Fluor 568 (Life Technologies, U.S.) labeled VHH (1 mg/ml) was added to the fixed cells in duplicate and incubated for 2 h at room temperature. Finally, the plates were observed with a Leica DMI4000 B microscope (Leica Microsystems, Buffalo Grove, IL), and fluorescent images were captured with a QImaging Retiga-2000R camera (Surrey, BC, Canada).

### Surface Plasmon Resonance (SPR)

Interaction studies between the VHHs and the NoV VLPs were performed using BIAcore X optical biosensors equipped with research-grade CM5 (for all the experiments) (GE Healthcare, U.S.) and CM3 (only for competition assays of M1 and M5 VHH) (GE Healthcare, U.S.) sensor chips. A standard coupling protocol was employed to immobilize either VLPs directly by amine coupling on the sensor or by capture with a coupled VHH M6 (for MD2004) or 7.3 (for GI.1 VLPs) [64]. For the direct coupling of the VLPs to the sensor, 10 μg/ml VLPs suspension (MD2004, Norwalk or P7-587) was diluted with 10 mM sodium acetate pH 5.0 and tested for pre-concentration in both CM3 and CM5-dextran surfaces. The capture surfaces were prepared by diluting the capture VHH to 10 μg/ml in 10 mM sodium acetate pH 5.0. The immobilizations were performed at 25°C using 0.005% Tween 20-PBS pH 7.4 (running buffer). One of the two flow cell CM-dextran surfaces was activated by a 7-min injection (5 μl/min) of freshly prepared 1:1 50mM NHS:200mM EDC [65]. Then, 35 μL of VLP or VHH (pH 5.0) were injected for 7 min at the same flow. This coupling was followed by a 7-min injection of 1M ethanolamine. The surfaces were immediately conditioned by three 6 sec injections of 50mM H_3_PO_4_. Approximately 3000 RU MD2004 VLPs were coupled on the CM3 sensor chip while 3000 RU VHH M6 was coupled on the CM5 surface. In both assays a second surface was kept intact and used as a reference for non-specific binding. Under these conditions, no response of VHH binding was recorded on the reference surface.

The binding analysis was performed at 25°C in running buffer and a data collection rate of 2.5 Hz. Higher throughput kinetic determination relied on screening of VHH fragments binding, using medium-throughput protocols previously described [66,67] and were performed at two concentrations of VHH (50 and 450 nM) to establish the kinetic constants and Rmax for each interaction. In a higher-resolution binding assays, VLPs were captured to 3000 RU, for GII.4 VLP and 300 RU for GI.1 VLPs. At varying concentrations of 0 (buffer), 6, 18.5, 55.6, 166.7 and 500 nM VHH samples were injected over all surfaces. The associations were monitored for 2 min, and the dissociations were monitored for 3 min or until 5% of the bound complex was dissociated [68]. In each cycle of the VHH M1 or M5, surfaces were regenerated with two 3-sec pulses in 50mM phosphoric acid. The binding responses were double-referenced [69]; VHH binding data were fit globally to a 1:1 interaction model (Biaevaluation software). The error of the fit to the model as well as the error for constants obtained in 2 or more binding assays are presented as standard deviation (SD) of the mean.

Competition assays were performed with directly immobilized MD2004 (3600 RU) by the method of sequential binding; briefly, VHH M1 was first bound at 450 nM, followed by VHH M5 at increasing concentrations spanning 0, 5.6, 16.7, 50 nM and 150 nM; the controls for maximum binding of VHH M5 were run at the same concentrations with buffer instead of VHH M1. A symmetric assay was also run by binding VHH M5 at 450 nM first, followed by VHH M1. The same competition study was performed for VHH N1 and N5 with the Norwalk VLPs.

### Peptide scanning for epitope mapping

An overlapping peptide library of 67 peptides corresponding to the P domain of the VP1 amino acid sequence from the NoV GII.3 Toronto strain was used to map the epitope specificity of VHH M6. The peptides were 17 amino acids long, with 12 amino acids overlapping with the previous and the next peptide of the sequence. Ninety six flat bottom well Neutravidin coated plates (Pierce Thermo Scientific, U.S.) were incubated with each biotinylated peptide in duplicate (2 μl of peptide/100 μl of PBS 0.1% BSA per well) ON at 4°C. After washing the plates, 100 ng/well of VHH M6 was added and the plates incubated for 2 h at room temperature. The plates were washed and incubated with rabbit anti-VHH serum at a 1:8,000 dilution for 1 h at room temperature. Finally, the plates were washed and then incubated with HRP-conjugated goat anti-rabbit IgG (H+L) (074–1516, KPL) at a 1:2,000 dilution for 1 h at room temperature. The assay was developed with commercial ABTS (50-66-18, KPL)/H_2_O_2_ substrate, added at 100 μl/well. The absorbance at 405 nm was read in an ELISA reader (Multiskan EX, Thermo scientific).

### Site-directed mutagenesis for epitope mapping

An alignment of the VP1 amino acid sequences of the MD2004 and MD145 strains identified variation in the residues exposed on the capsid surface. The pCI-MD2004 vector described previously [40] was modified using a Quik-change site-directed mutagenesis kit (Stratagene, Agilent Technologies, U.S.) and complementary forward and reverse primers to introduce MD145-specific nucleotide mutations into the capsid coding sequence. The restriction enzyme DpnI (10 U/μl) was used to digest the parental DNA. Each of the mutated products was transformed into the XL1-Blue supercompetent cells provided with the mutagenesis kit. Transformed cells were grown ON in LB plates with carbenicillin (50 μg/ml), and individual colonies were used for plasmid amplification. The resulting plasmids were subjected to sequence analysis to verify the entire VP1 coding region and confirm the presence of the introduced mutations. The mutation sites were selected according to the different surface amino acids between MD2004 and MD145 strains. The constructs were designated “MD2004 G340A”, “MD2004 E376Q”, “MD2004 N368T”, “MD2004 V389I”, and “MD2004 V340/E376”.

MD2004 mutagenized VP1 proteins were expressed in Vero cells as described above. After fixation in methanol, the monolayer was incubated with M7 VHH and binding of the VHH was detected incubating the plates with rabbit anti-VHH polyclonal serum, at a 1: 1,000 dilution for 1 h at room temperature. Finally, the plates were incubated with goat anti-rabbit IgG (H+L) conjugated with Alexa Fluor 488 (Life Technologies-Thermo Scientific, U.S.) at a 1:100 dilution for 1 h at 37°C and visualized under a fluorescence microscope. The wells were scored for positive and negative fluorescence, with a negative signal implicating a role for the mutagenized amino acids in VHH M7 epitope recognition. A conventional cross-reactive MAb, TV20, previously described [70], was included as a positive control to detect all the VP1 constructs.

MD2004 VLP containing amino acid substitutions V340/E376 was expressed and purified as previously described [40] and binding of VHH was detected by ELISA as described above.

### Bioinformatics analyses

Nucleotide sequences from the prototype strains were downloaded from GenBank and the deduced amino acid sequences were aligned. The solved structure of the P domain of VA387 virus (GII.4) in complex with carbohydrate (Protein Data Bank [PDB] accession number 2OBT) was used to identify the residues involved in binding with MAbs and was visualized by using UCSF Chimera software.

## Results

### Llama immunization

**Fig 1:**
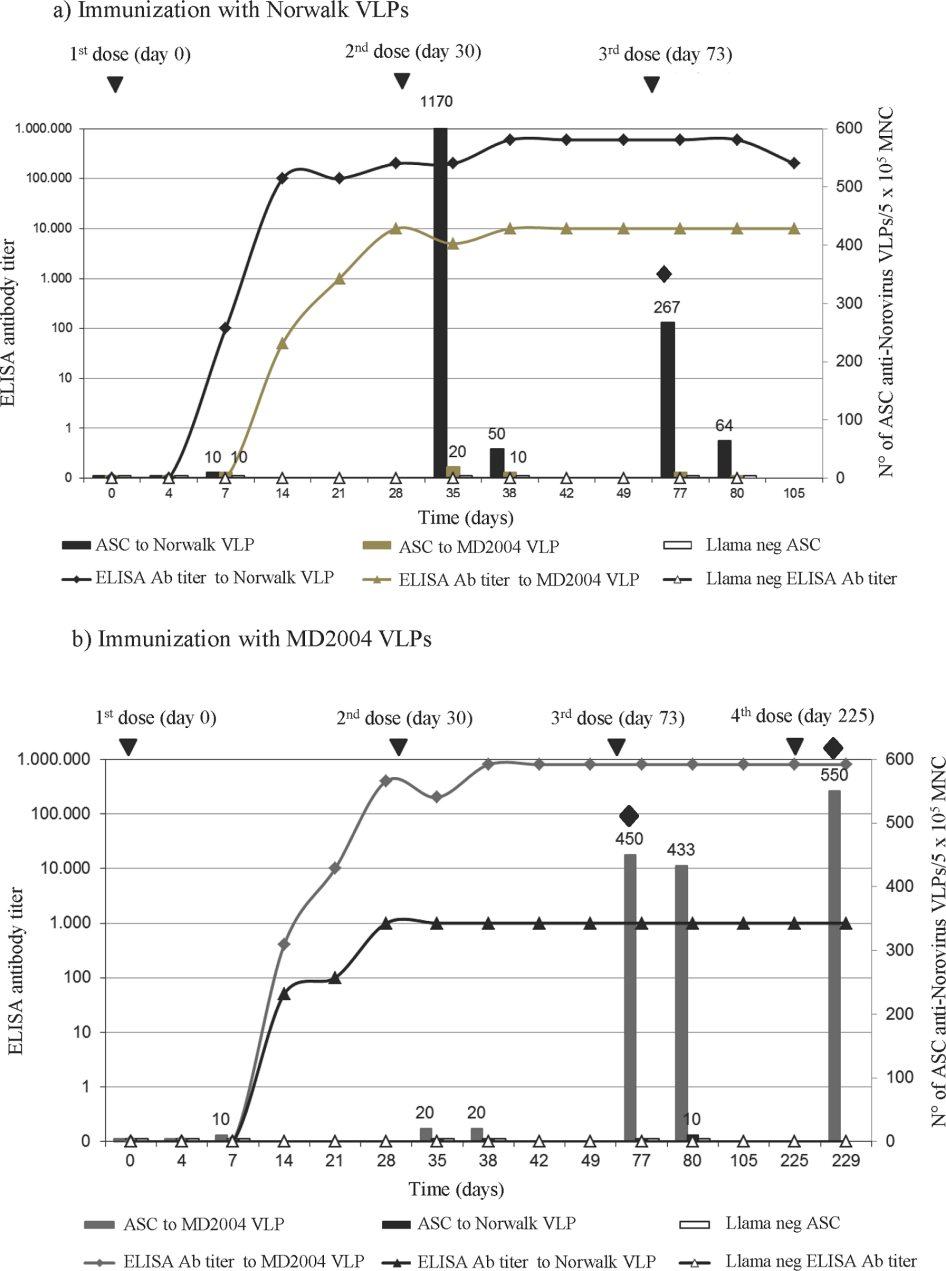
Llama immunization. The schedule for immunization (inverted triangle), sample collection and final bleeding (rhombus) is shown. The evaluation of NoV Ab response in serum during the time course of immunization is depicted for: a) Norwalk and b) MD2004. Antibody titers were measured by ELISA (lines) and the number of ASC for each llama were measured by ELISPOT (bars) using recombinant NoV VLPs Norwalk and MD2004.

With the aim of the construction of two VHH libraries, two male llamas were immunized with NoV VLP from the Norwalk virus strain (Hu/NoV/GI.1/Norwalk/ 1968/U.S.), or the MD2004 strain (Hu/NoV/GII.4/MD2004/2004/U.S.), respectively. The serum antibody (Ab) response to immunization measured by ELISA and the specific antibody secreting cell (ASC) response in peripheral blood measured by ELISPOT are depicted in Fig 1. Both llamas were seronegative for Ab to human NoV GI.1 and GII.4 prior to immunization. The llama immunized with Norwalk GI.1 NoV VLPs developed a high ELISA Ab titer against the homologous GI.1 VLPs that appeared at day 14 after the first dose and that reached a plateau at the 1:100,000 serum dilution after the second dose of antigen. A cross-reactive serum Ab response to MD2004 GII.4 VLPs was detected also in the GI.1 llama, but was of lower magnitude, reaching a plateau at the 1:10,000 serum dilution (Fig 1A).

The animal immunized with MD2004 GII.4 NoV VLPs developed a strong Ab response by ELISA to the homologous GII.4 antigen by day 14 reaching a plateau at the 1:800,000 serum dilution. This llama also showed seroconversion to Norwalk GI.1 VLPs but of lower magnitude reaching a plateau at the 1:1,000 serum dilution (Fig 1B). Seroconversion to the homologous, but not to the heterologous VLP, was detected utilizing the HAI assay (data not shown), suggesting that the cross-reactivity detected by ELISA would not be neutralizing.

Antibody secreting cell responses in the llamas were assessed against Norwalk and MD2004 NoV VLPs. These results showed the presence of high numbers of ASC to the homologous VLP (around 1000 and 500 ASC per 500,000 mononuclear cells (MNC) for Norwalk and MD2004 respectively) and approximately 1–2% of ASC with cross-reactivity (Fig 1A–1B).

After confirming optimal Ab titer and ASC responses, both llamas received a third immunization and were bled 4 days after the third dose. Additionally, the llama immunized with MD2004 VLPs received a fourth dose of antigen and was again bled four days later. An ELISPOT immunological parameter was used to select the proper time of the final bleeding for the library construction. A total of 450 ml of blood was obtained from the llama immunized with Norwalk VLPs yielding 5.91 x 10^8^ MNC, while 600 ml of blood extracted from the llama immunized with MD2004VLPs yielded 6.6x10^8^ MNC. From the processed total RNA, VHH fragments were amplified with specific primers, cloned into a phagemid vector and finally two VHH phage display libraries containing 4.2x10^8^ clones for Norwalk NoV and 1.0 x 10^8^ clones for MD2004 NoV were generated. Both VHH libraries possessed optimal size (in the order of 10^8^) according to published criteria [71].

### Phage display selection of VHH specific to Norwalk or MD2004 NoV strains

To select phages displaying VHH specific for each NoV genogroup, two rounds of *in vitro* selection (biopanning) were performed using the homologous NoV VLPs used in that llama vaccination as antigens. Furthermore, with the aim of obtaining highly specific VHHs to the immunizing antigen, two rounds of an extra biopanning strategy were performed as follows. The phages from each library were pre-incubated with the heterologous antigen to subtract the cross-reactive clones and the unbound phages were then incubated with the homologous antigen. After the second round of biopanning, 96 clones for each biopanning strategy (a total of 384 clones) were expressed in the context of the M13 phage particle as a fusion protein with the structural protein PIII and their ability to recognize NoV was tested by phage ELISA.

Ninety out of 96 clones from the VHH library specific for Norwalk virus recognized Norwalk VLPs, when the library was enriched twice with the homologous VLP. In the biopanning strategy where cross-reactive clones were subtracted by pre-incubation with MD2004 VLPs, the library became enriched in Norwalk-specific clones, with 32/96 phages recognizing Norwalk VLP by phage ELISA at the end of the second biopanning round.

For the MD2004 specific library, biopanned with the homologous antigen, 31/96 phages recognized MD2004 VLPs in the phage ELISA. When a prior subtraction step with Norwalk VLPs was performed to reduce cross-reactive phages, 44/96 clones recognized MD2004 VLPs. For all the tested phages, no reaction was observed in the blank plates or in the plates coated with the heterologous VLP. From the total positive clones obtained in each initial biopanning condition, the ones with the highest positive signal in phage ELISA were selected. From these clones, 18 VHHs with different amino acid sequences were successfully subcloned, expressed in the pHEN6 expression vector, and purified by His-tag. These included 10 VHH (N1-N10) derived from the Norwalk library and 8 VHHs derived from the MD2004 library (M1-M8) (Table 1).

**Table 1:** Selection of VHH for characterization.

Antigen of immunization	Antigen of selection in the biopanning	Antigen detected by Phage ELISA	Clone
Norwalk			N1
			N2
			N3
			N4
	Norwalk	Norwalk	N5
			N6
			N7
			N9
	Norwalk		N8
	(after substraction of cross-reactive clones with MD2004 VLP)	Norwalk	N10
MD2004			M1
			M2
			M3
	MD2004	MD2004	M4
			M5
			M6
	MD2004 (after substraction of cross-reactive clones with Norwalk VLP)	MD2004	M7
			M8

The VHH specific for Norwalk are named as N1-N10 while VHH specific for MD2004 are named as M1-M8.

### VHH domain specificity and reactivity in Western blot

Chimeric VLPs (the Norwalk S domain/MD2004 P domain chimera VLP and the MD2004 S domain/Norwalk P domain chimera VLP) [40] were used to determine the VP1 domain binding specificity of each VHH. All the VHH clones selected recognized the P domain of VP1 of the immunizing VLP present in the chimeric virus (Table 2). A WB assay was performed to assess whether the VHH could recognize denatured VP1. All VHH, with the exception of GII.4 clone M6, failed to recognize the VP1 protein in WB, suggesting that they are directed to conformational epitopes of the P domain. In contrast, clone M6 recognized the VP1 of MD2004 strain as well as that of other GII NoV strains belonging to different genotypes in WB (S1 Fig).

**Table 2:** Specificity of the VHHs to the P or S domain of VP1

VLPs	VHH GI.1 specific										VHH GII.4 specific							
	N1	N2	N3	N4	N5	N6	N7	N8	N9	N10	M1	M2	M3	M4	M5	M6	M7	M8
Norwalk (GI.1)	**+**	**+**	**+**	**+**	**+**	**+**	**+**	**+**	**+**	**+**								
MD2004 (GII.4)											**+**	**+**	**+**	**+**	**+**	**+**	**+**	**+**
Chimeric GII.4 (S) / GI.1 (P)	**+**	**+**	**+**	**+**	**+**	**+**	**+**	**+**	**+**	**+**								
Chimeric GI.1 (S) / GII.4 (P)											**+**	**+**	**+**	**+**	**+**	**+**	**+**	**+**
Binding Domain	P	P	P	P	P	P	P	P	P	P	P	P	P	P	P	P	P	P

In this table, (+) indicates a positive result, (-) a negative result and P indicates that the VHH binds within the P domain of VP1 protein. Chimeric GII.4 (S) / GI.1 (P) possess the S domain of MD2004 strain and the P domain of Norwalk strain while Chimeric GI.1 (S) / GII.4(P) possess the S domain of Norwalk strain and the P domain of MD2004 strain.

### VHH recognition of a diverse NoV VLP panel

The purified VHH were tested by ELISA against the NoV VLPs used in the immunization and biopanning as well as a panel of 26 VLPs representing different genogroups/genotypes of NoV (Table 3).

**Table 3:** Reactivity of the VHHs against VLPs representing strains from different genotypes.

Norovirus Strain	Year of Detection	Genotype	GI.1-specific VHH (ng/well)										GII.4-specific VHH (ng/ml)							
			N1	N2	N3	N4	N5	N6	N7	N8	N9	N10	M1	M2	M3	M4	M5	M6	M7	M8
**Norwalk 1968**	**1968**	**GI.1**	**0.50**	**0.50**	**1.00**	**0.50**	**0.50**	**1.00**	**1.00**	**2.00**	**0.50**	**1.00**	**-**	-	-	-	-	-	-	-
P7-587	2007	GI.1	0.50	0.50	1.00	1.00	0.50	1.00	1.00	-	0.50	-	-	-	-	-	-	-	-	-
Desert-Shield395	1990	GI.3	-	0.50	-	-	-	-	-	-	-	-	-	-	-	-	-	-	-	-
SzUG1	1997–99	GI.5	-	-	-	-	-	-	-	-	-	-	-	-	-	-	-	-	-	-
Hesse	1997	GI.6	-	-	-	-	-	-	-	-	-	-	-	-	-	-	-	-	-	-
Hawaii	1971	GII.1	-	-	-	-	**-**	**-**	-	-	**-**	-	-	**-**	**-**	1.00	1.00	1.00	-	1.00
Snow Mountain	1976	GII.2	-	-	-	-	-	-	-	-	-	-	-	-	-	2.00	2.00	2.00	-	2.00
Toronto 24	1991	GII.3	-	-	-	-	-	-	-	-	-	-	-	-	-	+	+	+	-	+
CHDC2005	2005	GII.3	-	-	-	-	-	-	-	-	-	-	-	-	-	+	+	+	-	+
CHDC5261	1990	GII.3	-	-	-	-	-	-	-	-	-	-	-	-	-	+	+	+	-	+
CHDC4031	1988	GII.3	-	-	-	-	-	-	-	-	-	-	-	-	-	2.00	0.50	1.00	-	0.50
Maizuru2000	2000	GII.3	-	-	-	-	-	-	-	-	-	-	-	-	-	+	+	+	-	+
Aus2001	2001	GII.3	-	-	-	-	-	-	-	-	-	-	-	-	-	+	+	+	-	+
Aus2007	2007	GII.3	-	-	-	-	-	-	-	-	-	-	-	-	-	+	+	+	-	+
Aus 2008	2008	GII.3	-	-	-	-	-	-	-	-	-	-	-	-	-	+	+	+	-	+
CHDC32	1976	GII.3	-	-	-	-	-	-	-	-	-	-	-	-	-	+	+	+	-	+
CHDC4871	1977	GII.4	-	-	-	-	-	-	-	-	-	-	0.25	-	0.50	0.50	0.50	2.00	-	1.00
Rockville	2012	GII.4	-	-	-	-	-	-	-	-	-	-	0.25	0.25	0.50	1.00	0.50	0.50	-	2.00
**MD2004**	**2004**	**GII.4**	-	-	-	-	-	-	-	-	-	-	**0.50**	**0.50**	**2.00**	**2.00**	**1.00**	**0.50**	**0.50**	**2.00**
MD145	1987	GII.4	-	-	-	-	-	-	-	-	-	-	0.50	8.00	1.00	2.00	4.00	0.50	-	4.00
HS191	2001	GII.4	-	-	-	-	-	-	-	-	-	-	1.00	0.50	0.50	0.50	0.25	2.00	-	1.00
Bethesda	2012	GII.6	-	-	-	-	-	-	-	-	-	-	-	-	0.50	2.00	1.00	1.00	-	2.00
DC119	1978	GII.7	-	-	-	-	-	-	-	-	-	-	-	-	-	0.50	0.50	1.00	-	1.00
M7	1999	GII.14	-	-	-	-	-	-	-	-	-	-	-	-	-	-	-	1.00	-	-
St. Cloud 624	1998	GIV.1	-	-	-	-	-	-	-	-	-	-	-	-	-	-	-	-	-	-

Reactivity of the VHHs against VLPs representing strains from different genotypes. A (-) sign indicates the absence of reaction, a (+) sign indicates a positive reaction and for the VLPs where quantitative assays were conducted the number inside de cell indicates the minimum amount of VHH/well with positive reaction.

The ten VHH clones obtained from the Norwalk library reacted with the homologous GI.1 VLP. All clones except N8 and N10 were able to detect the P7-587 strain, a 2007 GI.1 strain that circulated decades after Norwalk virus. Only clone N2 was able to recognize the Desert Shield 395 strain that belongs to the GI.3 genotype. None of these VHH reacted with GI.5 and GI.6 strains or with GII and GIV NoVs (Table 3).

The eight VHH clones derived from the GII.4 MD2004 library were able to detect the homologous VLP by ELISA. These clones displayed a variety of recognition patterns. Clone M7 reacted only with the GII.4 MD2004 strain. Clone M2 detected with high affinity 3 out of 5 GII.4 strains, clone M1 reacted with all GII.4 strains, clone M3 reacted with all GII.4 strains and in addition, with a GII.6 strain. Clones M4, M5 and M8 recognized GII NoV strains belonging to genotypes 1, 2, 3, 6 and 7. Finally, the clone M6, directed to a linear epitope, recognized all the GII VLPs tested by ELISA (Table 3). Clones N8 and N10, specific only for Norwalk virus, and clone M7 specific only for MD2004 virus (Table 3) (the antigens used for their respective llama immunizations) were selected using subtractive biopanning strategies (Table 1).

### Reactivity of VHH in surrogate virus neutralization assays: HBGA blocking and hemagglutination inhibition

**Fig 2:**
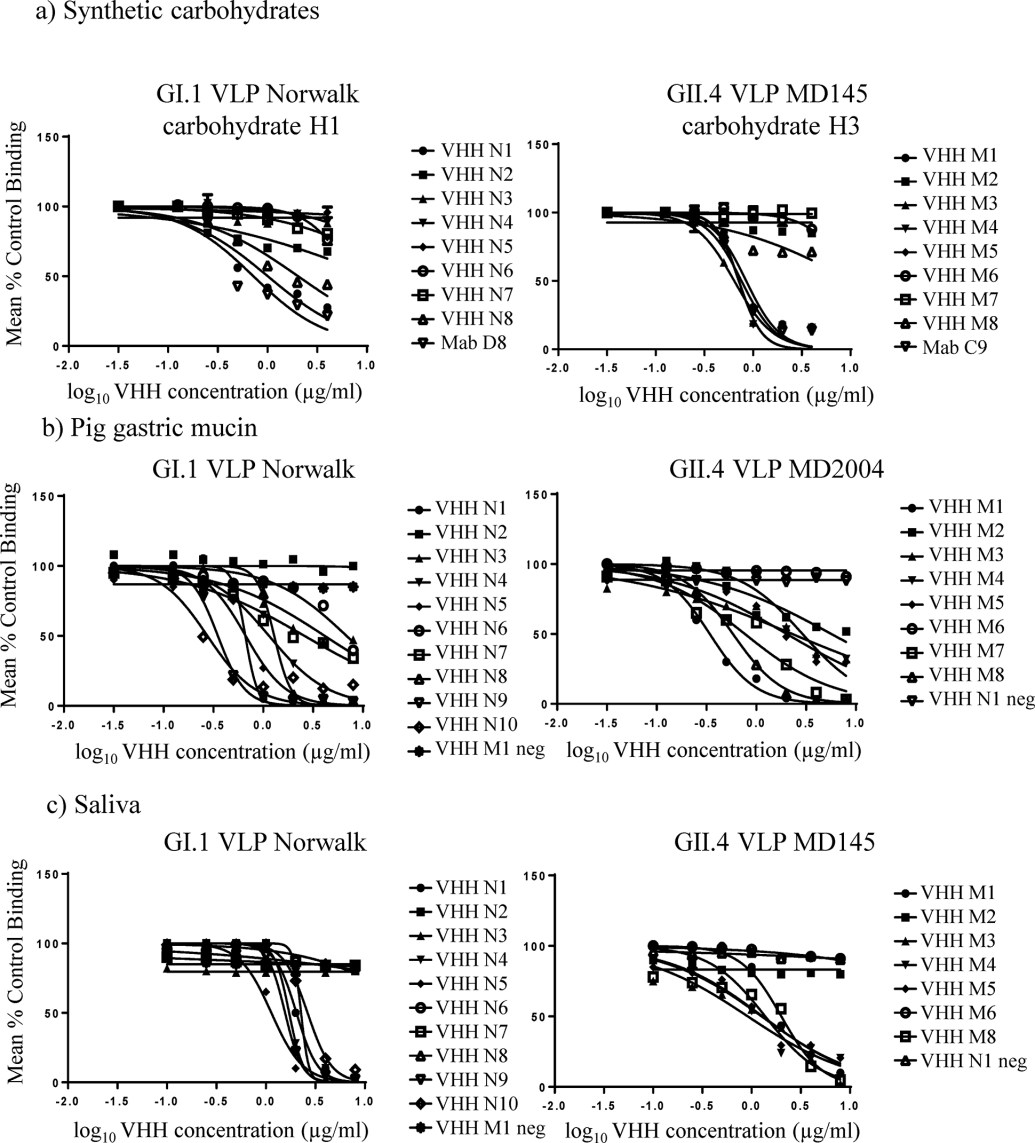
Blockade assays. Surrogate virus neutralization tests were performed using different sources of carbohydrates: (a) synthetic carbohydrates H1 for Norwalk GI.1 VLPs or H3 for GII.4 MD145 VLPs; (b), PGM type III for Norwalk GI.1 or MD2004 GII.4 VLPs and (c) saliva for Norwalk GI.1 or MD145 GII.4 VLPs. Sigmoidal curves were fit to the mean percent control binding calculated by comparing the amount of VLP bound to each source of carbohydrate in the presence of VHH pretreatment to the amount of VLP bound in the absence of pretreatment.

**Fig 3:**
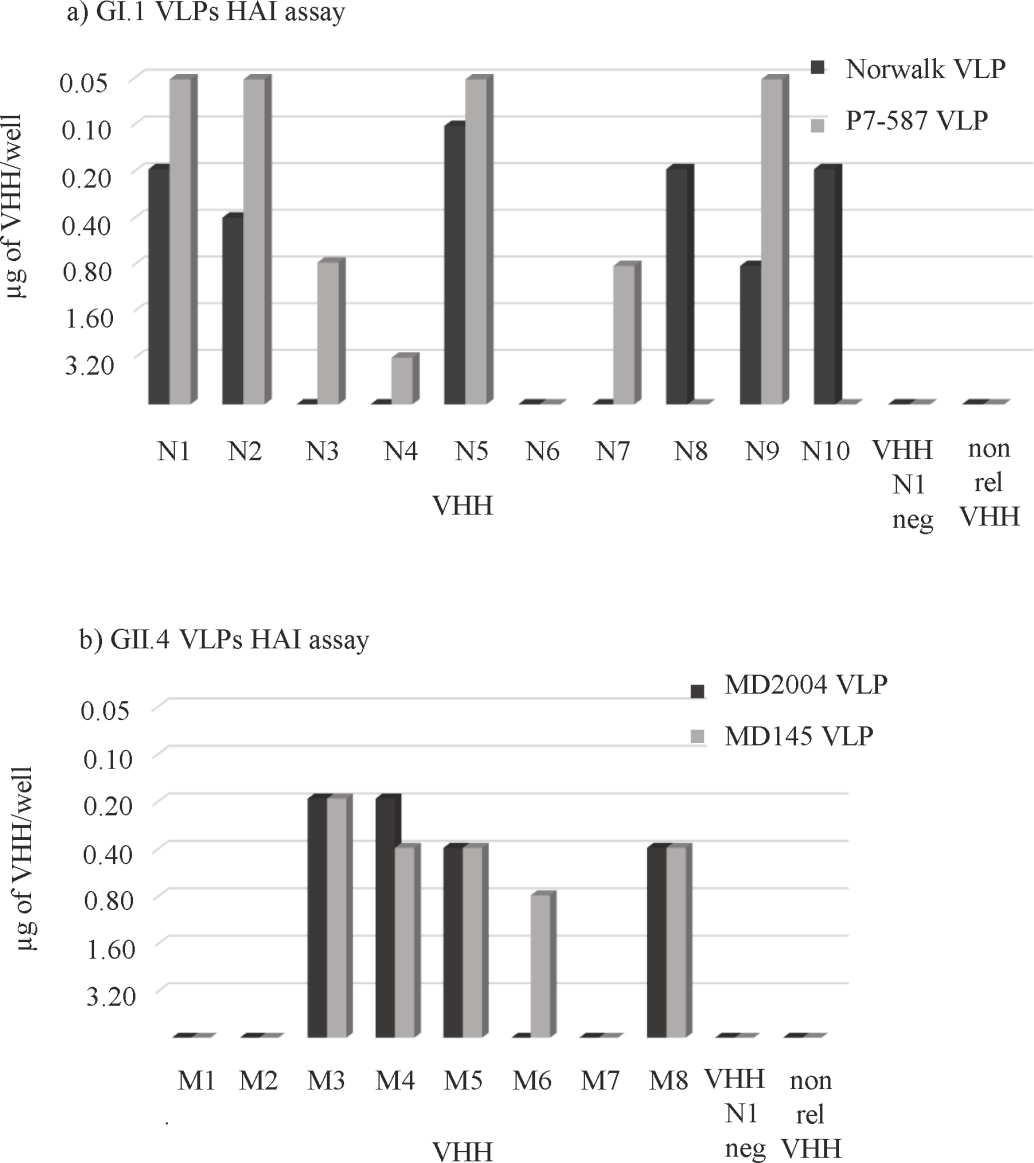
Hemagglutination inhibition (HAI) assay. The HAI titer of each VHH was defined as the lowest antibody concentration that completely prevented NoV VLP-mediated hemagglutination of human RBCs. Norwalk or P7-587 GI.1 VLPs hemagglutinated 0 Rh- RBC (a); MD2004 or MD145 GII.4 VLPs hemagglutinated B Rh+ (b).

Previous studies had noted a correlation between antibody HBGA blocking (blockade) and hemagglutination inhibition (HAI) titers with protection from norovirus disease [35,37], leading to their use as surrogate neutralization assays. The ability of small VHH molecules to inhibit the interaction of norovirus VLPs with HBGA carbohydrates was not known, so three different HBGA blocking (blockade) assays using synthetic HBGA molecules, pig gastric mucin, or saliva as carbohydrates were employed to examine this property. Also HAI assay was carried out. The results are illustrated in Figs 2 and 3 and summarized in Table 4.

**Table 4:** Summary of VHH activity in different surrogate neutralizing assays.

VHHGI.1		EC_50_ (μg/ml)		HAI titer (μg/ml)	
	HBGA H1	PGM type IIILey, A and H2 +	Saliva Ley+	Blood typ 0, Ley +	
	Norwalk	Norwalk	Norwalk	Norwalk	P7-587
	VLP	VLP	VLP	VLP	VLP
**N1**	**1.006**	**0.661**	**2.035**	**0.19**	**0.05**
N2	-	-	-	0.39	0.05
N3	-	4.139	-	-	0.78
N4	-	1.144	1.718	-	3.12
N5	-	0.667	1.136	0.1	0.05
N6	-	6.856	-	-	-
N7	-	2.594	-	-	0.8
**N8**	**2,032**	**1.289**	**2.267**	**0.19**	**-**
N9	ND	0.351	1.563	0.78	0.05
N10	ND	0.275	2.637	0.19	-
VHH GII.4		EC_50_ (μg/ml)		HAI titer (μg/ml)	
	HBGA H3	PGM type III	Saliva Ley+	B Rh+ RBC	
	MD145	MD2004	MD145	MD2004	MD145
	VLP	VLP	VLP	VLP	VLP
**M1**	**0.85**	**0.341**	**2.008**	-	-
M2	-	-	-	-	-
**M3**	**0.641**	**2.296**	**0.917**	**0.19**	**0.19**
M4	0.712	2.909	1.379	0.19	0.39
**M5**	**0.718**	**2.023**	**1.268**	**0.39**	**0.39**
M6	-	-	-	-	0.78
M7	-	0.767	-	-	-
M8	-	0.583	1.296	0.39	0.39

In this table (-) indicates no blocking activity and values indicate the concentration of VHH in μg/ml that caused 50% of blocking effect (EC_50_), and ND, non-determined. Bold letters showed the VHH with overall best blockade performance.

Seven out of the 10 VHH specific to GI NoVs showed a blockade property by one or more of the surrogate neutralization assays. The best performance was observed with clone N1 in that it could inhibit GI.1 VLP attachment to the carbohydrates in all the blockade assays and also showed HAI properties. VHH N5 performed well also because it could inhibit both Norwalk and P7-587 VLP binding to carbohydrates of the PGM, saliva and human RBC. Finally, clones N8 and N10 were effective but only for the Norwalk virus strain.

In the GII.4 MD2004 library, clones M3, M4 and M5 could inhibit GII.4 VLP binding to H3 carbohydrate, saliva (half maximal effective concentration (EC_50_) between 0.6 to 1.3 μg/ml) and showed good HAI activity (HAI titer between 0.19 to 0.39 μg/ml). However, slightly higher amounts of VHHs were needed to block the attachment of VLPs to the PGM (EC_50_ 2 μg/ml or higher). VHH GII.4 clone M1 was able to interfere with VLP binding to synthetic carbohydrate H3, PGM and human saliva, but failed to demonstrate HAI activity. Clone M8 was able to inhibit VLP attachment to PGM and saliva and showed HAI properties. Clone M7, was able to inhibit VLP attachment to PGM with a good EC_50_, but failed to block carbohydrate binding and did not show HAI activity. Finally, VHH M2 and M6 (that recognized a linear epitope) did not show blocking activity.

### Competition assays

**Fig 4:**
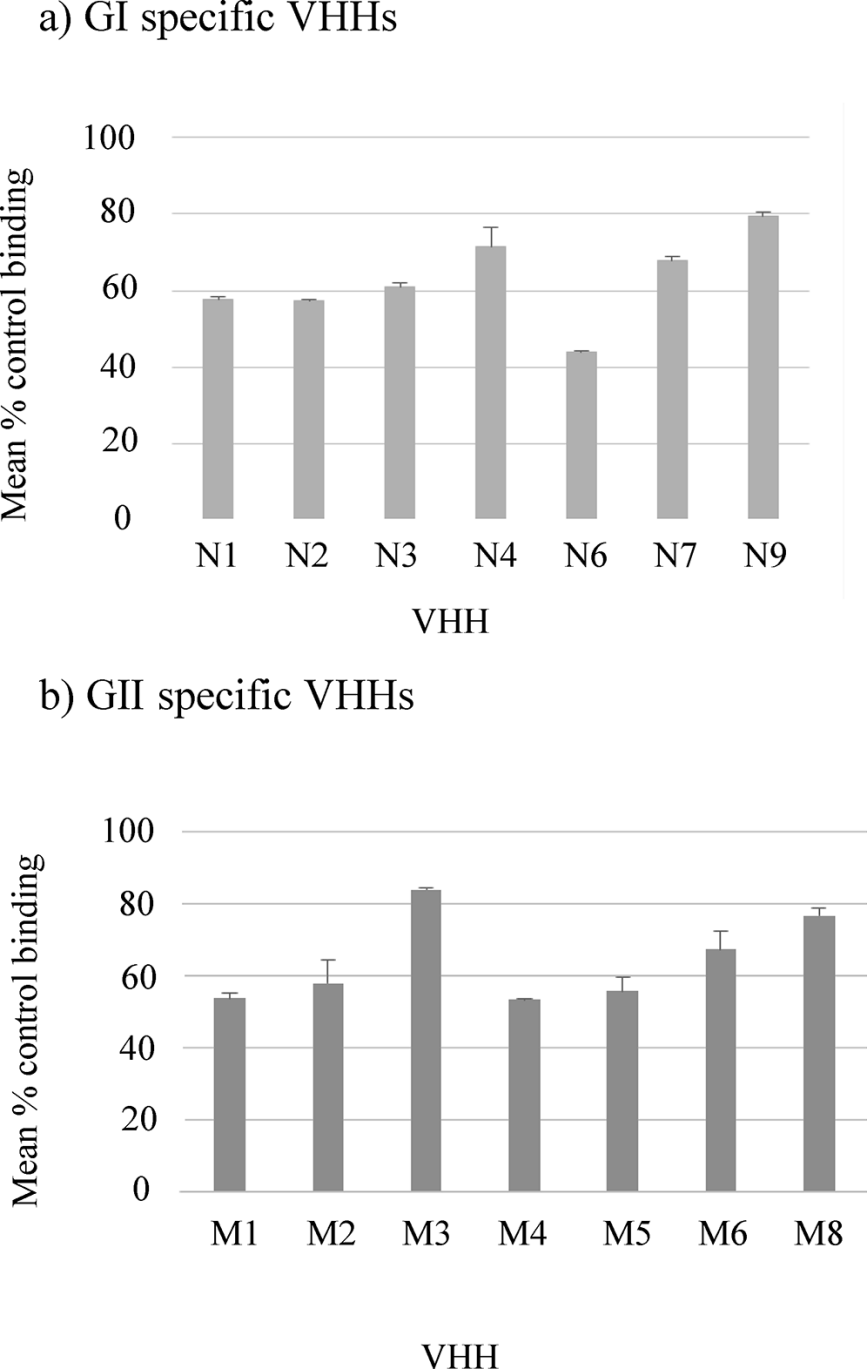
Hyperimmune llama serum blocking assay. The percentage of VHH binding to hyperimmune serum pretreated VLPs was calculated compared to the positive control of each VHH binding to the VLPs without pretreatment. Bars represent the average of two independent assays.

To evaluate the ability of a polyclonal serum to compete with VHH binding to NoV MD2004 or Norwalk VLPs, competitive binding assays were performed. When llama NoV strain-specific hyperimmune serum was used to compete with VHH binding to VLPs, the binding of 6.5 ng/well of any VHHs to the corresponding VLP was reduced between 47% and 16% (Fig 4). The only exception was the binding of the VHH N6 to GI.1 Norwalk VLP that was reduced 56%. This result indicates that most of the VHH clones were able to bind to their corresponding epitope within the P domain of the VLP even in the presence of a high concentration of conventional antibodies in polyclonal serum including those likely directed to the same epitopes. The reciprocal competition assay indicated that pre-incubation of the VLPs with high amounts of VHH did not impair the binding of the hyperimmnune serum to any of the VHH or combinations of VHH tested.

Given the results obtained in the surrogate neutralization assays, two VHH specific for each NoV genogroup were selected and labeled with Alexa Fluor 568 dye for analysis in immunofluorescence (IF) competition assays in order to determine whether their cognate epitopes overlapped. The results showed that GI.1 clones N1 and N5 did not compete with each other nor with any other GI specific VHH for binding to NoV GI.1 VP1 expressed in cells, and would be thus directed to different epitopes.

**Fig 5:**
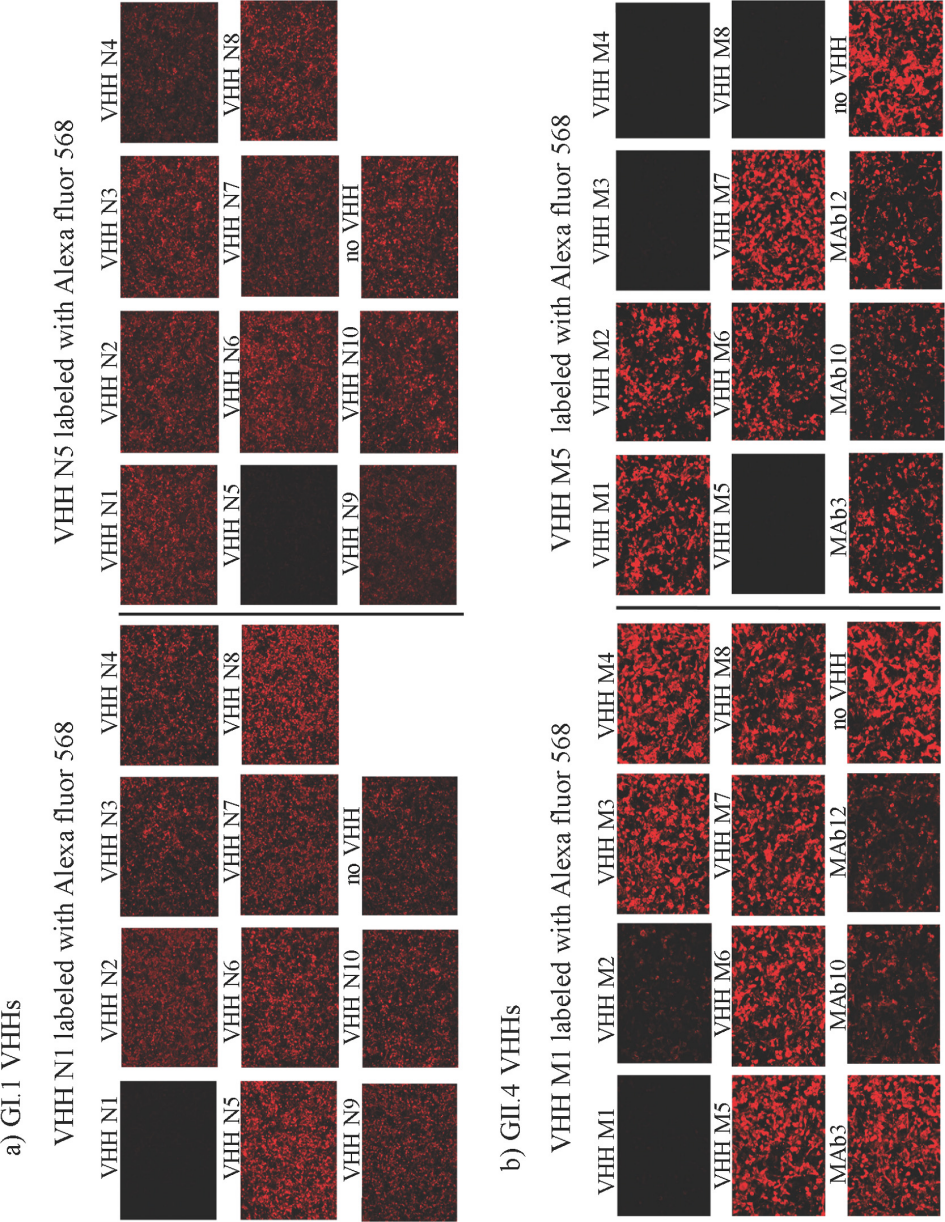
Competition assay between VHHs. The images show immunofluorescence staining of Vero cells expressing NoV VP1 from Norwalk strain (a) or MD2004 strain (b). Ten μg of an unlabeled VHH together with 1 μl of Alexa Fluor 568 labeled VHH were incubated on the fixed cells. A decrease in the fluorescent signal indicates a competitive binding with the labeled VHH.

Regarding the GII specific VHHs, clones M1 and M5 recognized non-overlapping epitopes. Furthermore, VHH M1 did not compete with any other GII.4 VHH in the panel tested, confirming that it was a unique epitope. In contrast, the binding of labeled VHH M5, was inhibited by the presence of VHH M3, M4 and M8 in the competitive IF assay, suggesting that all these clones may be directed to the same epitope (Fig 5). This observation is in agreement with similarities detected in the CDR3 region sequences (data not shown). VHH M1 and M5 clones were not competed by murine-origin conventional GII.4-specific MAbs 3, 10 and 12 that we reported previously [40].

### Affinity measurements by Surface Plasmon Resonance (SPR)

We next examined the representative VHH pairs for GI (VHH N1 and N5) and GII (VHH M1 and M5) by SPR to confirm the competition results above and to evaluate their affinity. The GI VHH clones N1 and N5 showed affinities to immobilized Norwalk VLP of 1.1 and 33 nM respectively (Table 5) and VHH N5 showed a higher affinity for P7-587 VLPs of 9.7 nM (Table 5). As expected, the competition assays did not show any interference or competition between these two binders, as the kinetic profile for each VHH did not change with the previous addition of the other VHH (data not shown).

**Table 5:** Kinetic constants for different VHH determined by Biacore binding assays.

VHH	k_a_	k_d_	K_D_ (95% CI)	VLPs
	(x 104 M-1 s-1)	(x 10–4 s-1)	nM	
M1	5.68±0.2	0.81±0.01	1.44 (0.5–2)	MD2004
M5	16.20±0.1	3.10±0.05	1.92 (1.5–3.5)	MD2004
M6	59.4±0.1	0.25±0.02	0.04 (0.03–0.05)	MD2004
N5	5.0±0.2	21.5±0.6	33.0 (30–35)	Norwalk
N1	3.0±0.3	35.0±0.7	1.17 (1–2)	Norwalk
N5	6.34±	6.15±	9.70 (7.9–10)	P7-587

Association rate constants (k_a_), dissociation rate constants (k_d_), equilibrium association constants (and equilibrium dissociation constants (KD = k_d_/k_a_). Independent experiments: n = 1, 2, 3. 95% CI expresses SD between independent experiments, otherwise, the SE of the fit was computed to estimate the apparent affinities for the interactions.

**Fig 6:**
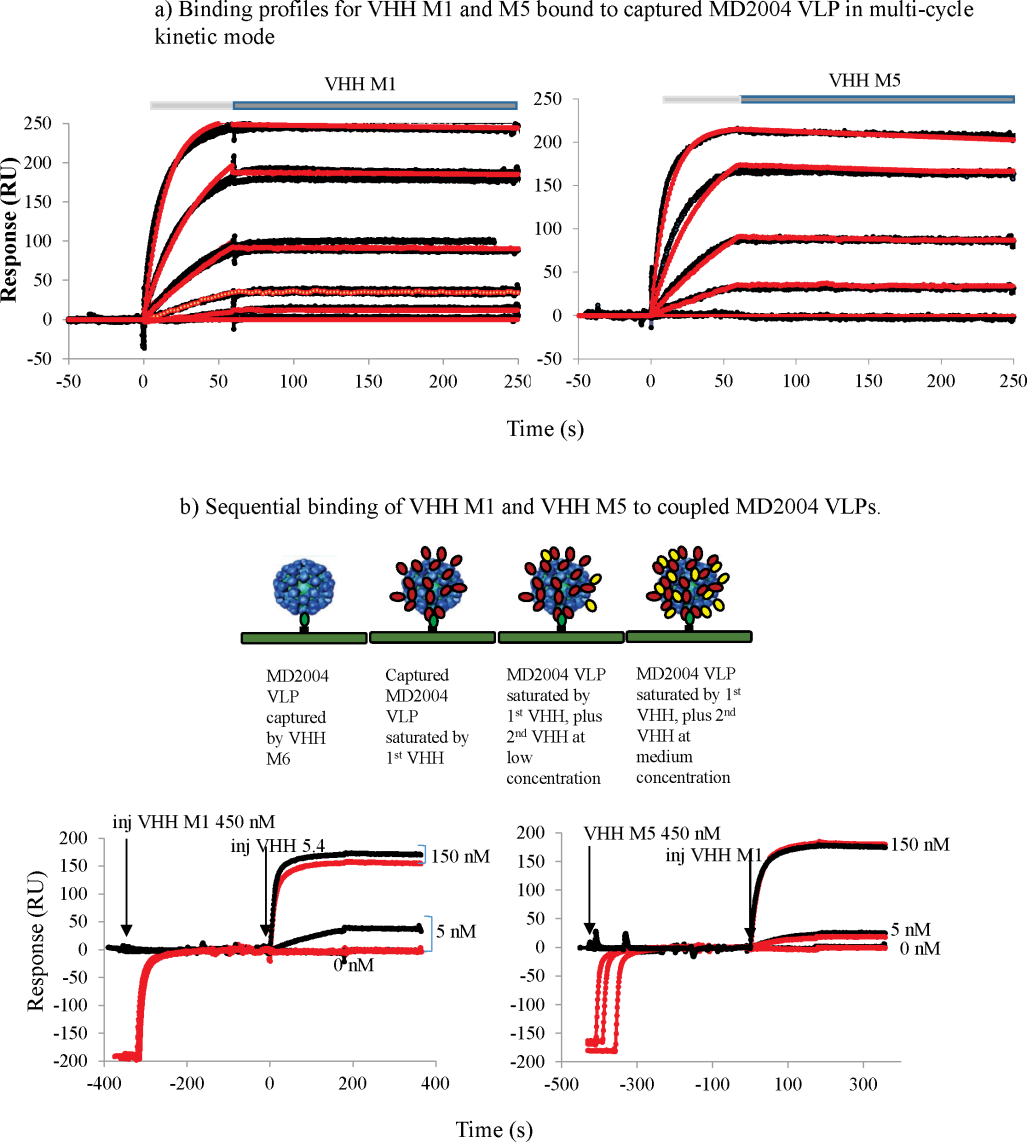
BIAcore binding assays. a) Association (light gray block) and dissociation (dark grey block) profiles for VHH M1 or M5 binding to MD2004 VLP coupled on the sensor surface. VHH M1 concentrations spanned from 45 to 1226 nM and VHH M5 concentrations spanned from 6 to 167 nM. The surface density of VLPs was 3000 RU for both VHHs. Red lines correspond to the fit of a 1:1 (one step) binding model. b) Sequential binding of VHH M1 and VHH M5 to coupled MD2004 VLPs. VHH M1 injected at 450 nM completely inhibited the binding of 5.57 nM. Inhibition was overcome with 150 nM VHH M5. VHH M5 was injected at 450 nM but did not modulate VHH M1 binding as shown in the case of the 5.57 or 150 nM injection of VHH M1. Contact times for both injections were 180 s. The black profiles correspond to the kinetics of binding of VHH (corresponding case) binding to MD2004 while the red profiles highlight the binding profile in the presence of VHH.

The GII VHH clones M1 and M5 showed affinities around the low nanomolar range for captured VLP MD2004, with equilibrium dissociation constants of 1.4 and 1.9 nM respectively. VHH M5 had a faster off-rate, and VHH M1 had a faster on-rate showing opposite behaviors but with similar overall affinity (Fig 6A).

Competition assays performed with directly immobilized MD2004 by the method of sequential binding indicated that no competition or interference was observed for the binding of low or high concentrations of VHH M1 when VHH M5 was previously bound in excess to the MD2004 VLP. However, some interference was observed for the binding of VHH M5 in low concentrations when VHH M1 was previously bound saturating its epitopes in the MD2004 VLP. This observed interference minimizes at high concentrations of VHH M5 (Fig 6B).

Interestingly, the VHH M6 showed cross reactivity with all the GII VLPs tested (GII.1, GII.2, GII.3, GII.4, GII.6, GII.7, and GII.14) and would be an excellent reagent for the detection of this prevalent NoV in fecal samples. The SPR analysis showed that this VHH possessed very high affinity in the picomolar range, with a KD of 40 pM (Table 5), higher than the other VHH tested in this work, and beyond the affinity ceiling of 100 pM that has been suggested for antibodies generated through in vivo maturation [72–74].

### Mapping the linear epitope recognized by GII VHH M6

The GII VHH M6 recognized all the GII strains tested by WB (S1 Fig) and ELISA (Table 3) indicating that it was directed to a conserved linear epitope within the P domain of VP1 (Table 2). It also possessed a high affinity in the picomolar range.

**Fig 7:**
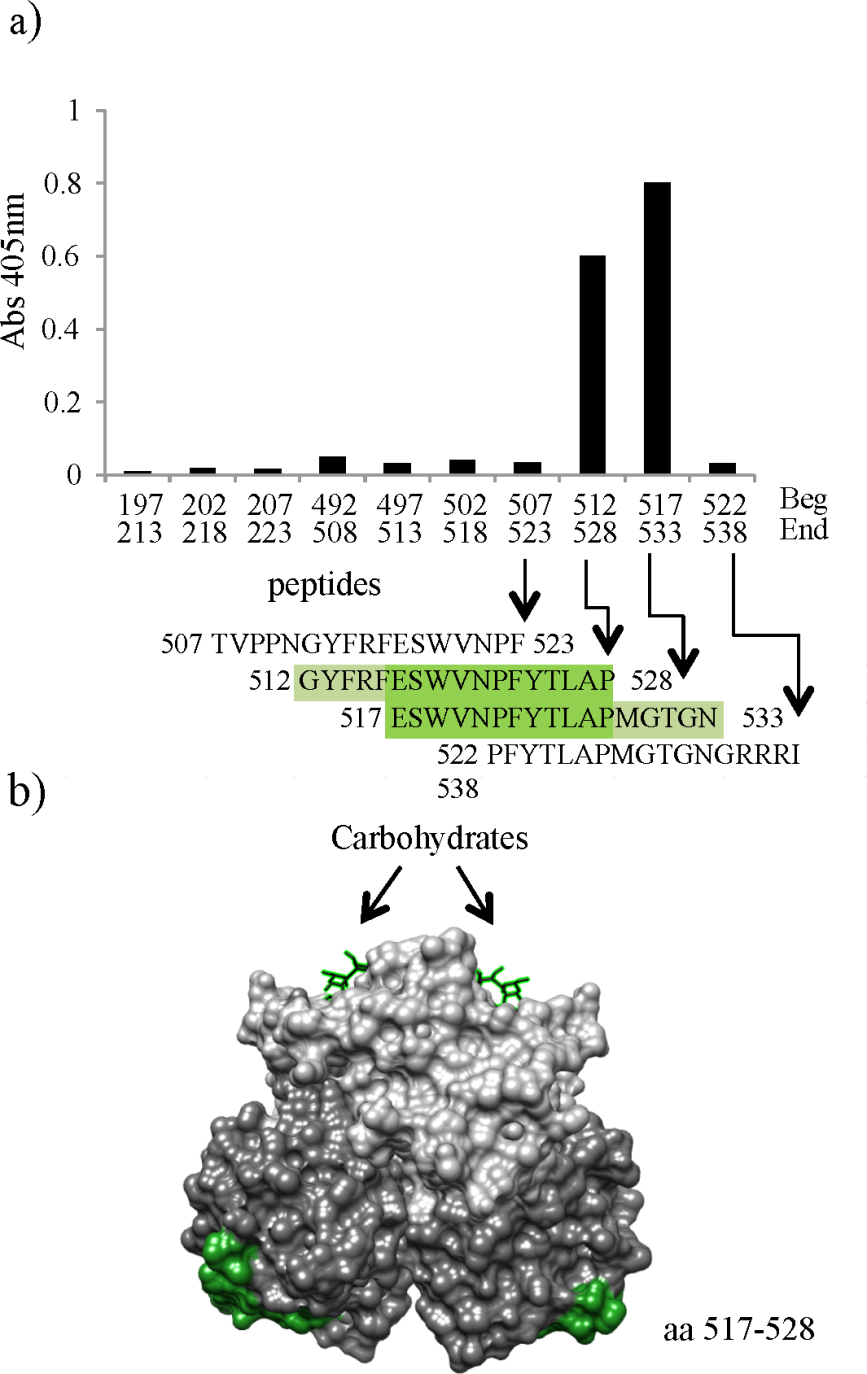
Epitope mapping of VHH M6. a) ELISA with overlapping peptides corresponding to the P domain of VP1 NoV. b) Putative linear epitope of VHH M6 shown in the structure of a GII.4 P dimer. Subdomains of norovirus VP1 are represented in different colors, P1 in light grey and P2 in dark grey. The putative M6 epitope is shown in green.

To map the epitope recognized by VHH M6, an available peptide library corresponding to the P domain of the VP1 amino acid sequence from the GII.3 NoV Toronto strain was utilized. The library consisted of 67 peptides that were each 17 amino acids long, with 12 amino acids overlapping with the previous and the next peptide of the sequence. The VHH M6 was tested against each of the peptides and a positive reactivity was obtained for the overlapping peptides corresponding to amino acids 512–528 (GYFRFESWVNPFYTLAP) and 517–533 (ESWVNPFYTLAPMGTGN) while no reactivity was observed for the flanking overlapping peptides 507–523 (TVPPNGYFRFESWVNPF) and 522–538 (PFYTLAPMGTGNGRRRI) (Fig 7). These data ruled out sequences GYFRF and MGTGN as essential for formation of the epitope. Thus, the putative epitope would be located within the overlapping sequences of the two positive peptides, specifically within amino acids 517 to 528 (ESWVNPFYTLAP). This region of the P domain of VP1 protein is located near the C-terminal end of the VP1 protein (Fig 7B) and is highly conserved among GII NoVs.

### Mapping the conformational binding epitope recognized by GII VHH M7

GII.4 VHH clone M7 was highly specific for the VP1 of the MD2004 strain and did not recognize other GII strains, including GII.4 MD145. A panel of MD2004 VP1 variant proteins bearing the amino acid substitutions in the P domain between these two strains was generated using the pC-MD2004 expression vector to gain insight into the identity of the recognized epitope. The generated constructs in pCI vector were designated “MD2004 G340A”, “MD2004 E376Q”, “MD2004 N368T”, “MD2004 V389I”, and “MD2004 V340/E376”. Vero cells were transfected with the pCI constructs and VHH M7 binding was detected with rabbit anti-VHH polyclonal serum and anti-rabbit IgG labeled with Alexa Fluor 488 dye.

**Fig 8:**
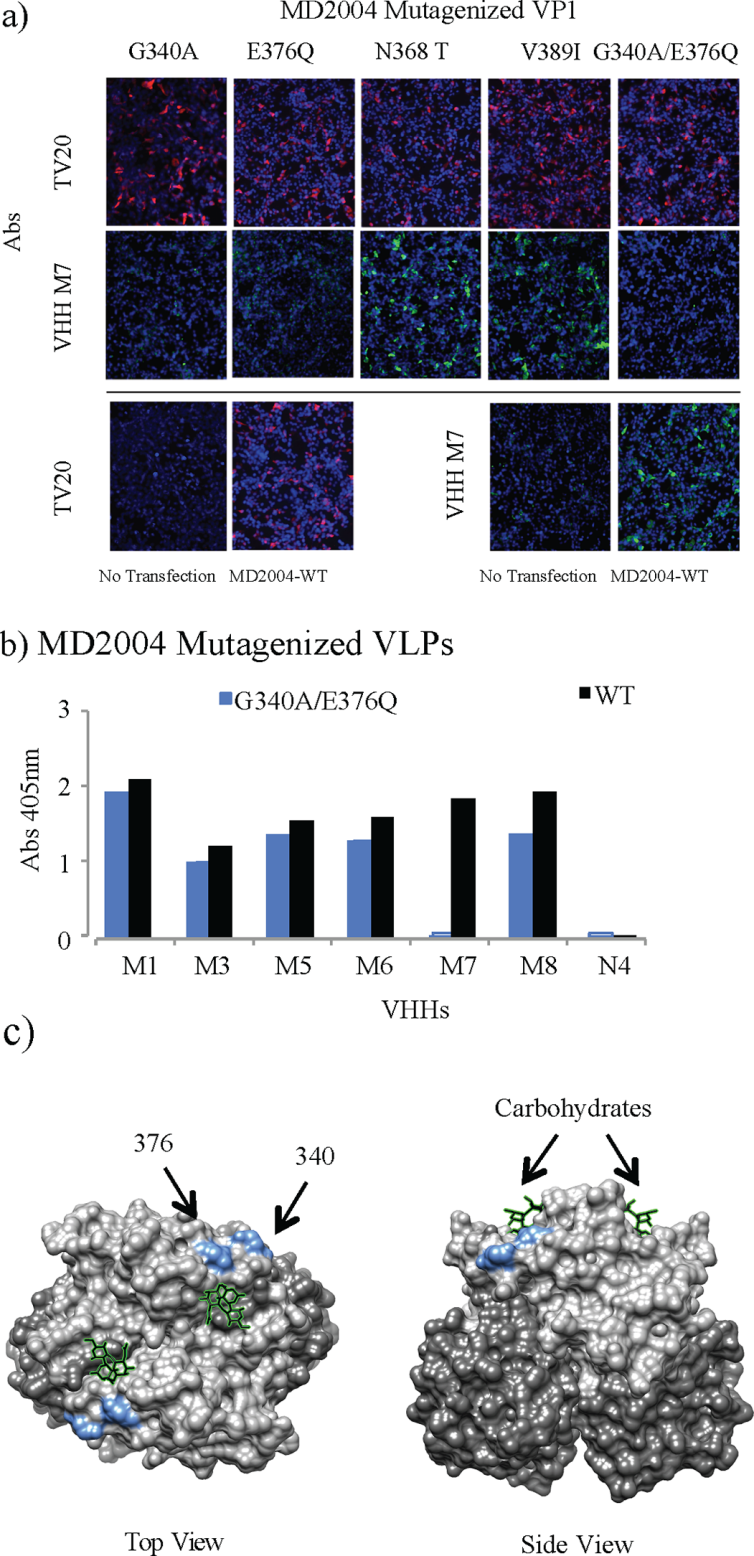
Epitope mapping of clone M7 specific for the GII.4 MD2004 strain. a) Expression of MD2004 VP1 variants in Vero cells. The images show immunofluorescence staining of Vero cells transfected with pCI constructs carrying VP1 from wild type MD 2004 and five P domain mutants: E376Q, G340A, N368T, V3891I, and V340/E376. VHH M7 binding was detected with rabbit anti-VHH polyclonal serum and anti-rabbit IgG labeled with Alexa Fluor 488 dye. Labeled MAb TV20 directed to the S domain was used as positive control. b) P domain structure showing the amino acids involved in VHH M7 epitope (light blue). Subdomains of norovirus VP1 are represented in different colors, P1 in light grey and P2 in dark grey.

The IF assay showed that mutations in amino acids 368 and 389 did not impair VHH M7 binding (Fig 8A), while VHH M7 failed to recognize a double substitution variant (G340A/E376Q) as well as the single substitution variants (G340A and E376Q) of VP1 indicating that both amino acids 340 and 376 are critical for the binding and may be implicated in the epitope (Fig 8A). In order to confirm the IF data, and present the epitope in structural context, recombinant VLPs representing the double escape mutant G340A/E376Q were developed, expressed, and purified. Of the anti-MD2004 VHH panel, only VHH M7 failed to recognize this modified VLP by ELISA (Fig 8B), in concordance with the IF assay.

## Discussion

Noroviruses (NoV) are recognized as a leading cause of viral gastroenteritis affecting all age groups. Additionally, they can cause life-threatening disease in the very young and the elderly. Immunocompromised patients are at risk because of chronic norovirus infections that can cause diarrhea that lasts from weeks to years [1,11]. Passive immune therapies based on specific polyclonal and monoclonal antibodies (MAbs) have recently been considered for NoV prevention and treatment [75]. Chicken egg yolk immunoglobulins directed to NoV and rotavirus have been developed and proposed as a potential combined therapy [76,77]. However, the immunogenicity of avian IgY and its potential to cause allergic or hypersensitivity reactions remain an important issue to consider before its use in the general population [78]. The application of MAbs more similar in structure to human IgG for the treatment of NoV diarrhea could lessen the risk for immunogenic reactions. Specific Fabs with strong HBGA blocking activity were shown to prevent Norwalk virus infection in chimpanzees when virions were pre-incubated with the Fabs prior to challenge [41]. In addition, conventional monoclonal antibodies directed toward a GII strain were developed with blockade properties that have potential for humanization [13,79]. More recently, from a phage display library of human synthetic single chain antibodies (scFv), several scFv specific to NoV were selected [42]. Two VHH antibodies specific for certain GII NoV strains were recently described and one showed the ability to disassemble NoV GII.4 and GII.10 VLPs, consistent with a possible antiviral activity in the host [80].

Llama-derived single chain antibody fragments, VHH, are the smallest molecules in nature with antigen-binding capacity and offer a number of advantages compared to conventional antibodies for therapeutic applications. In the present study, two VHH libraries against NoV GI.1 (Norwalk) and GII.4 (MD2004) were developed with the goal of isolating high affinity antibodies specific for the immunizing strains. After immunization, llamas developed a robust serum antibody response to the homologous NoV and a lower cross-reactive antibody response to the heterologous strain as measured by ELISA and ELISPOT. Seroconversion to the homologous, but not to the heterologous VLP, was detected utilizing the HAI assay (data not shown), suggesting that the cross-reactivity detected by ELISA would not be neutralizing. However, the detection of cross-reactivity is promising in that VHH phage libraries can be rescreened with innovative strategies to focus on broadly cross-reactive antibodies that might be protective.

The conditions utilized for the construction of the libraries in our study as well as the selection of the clones allowed the isolation of VHH that were highly specific for GI or GII. According to previous work, the Norwalk virus capsid is susceptible to disassembly at basic pH [81,82]. The screening of VHH at alkaline pH was preferred to promote the exposure of a wide range of epitopes due to the pH dependent conformational changes of the VLP particle [82]. Sequence analysis of the VHH clones positive for phage ELISA was subsequently performed (data not shown). A sequence variety was obtained as expected for both genogroups. However, regarding the CDR3 amino acid sequences of VHH specific for GII genogroup, from twenty sequenced clones, six different profiles were retrieved. This suggests that the strategy used for the VHH screening only allowed the selection of clones directed toward a limited number of epitopes [83,84]. A total of eighteen recombinant monoclonal nanobodies directed toward the P domain of the NoV VP1 capsid protein were selected for further analysis. One nanobody (VHH M6) was directed toward a linear epitope of the GII while seventeen VHH were directed toward conformational epitopes (10 VHHs specific for GI genogroup and 7 VHH specific for GII genogroup). Using a panel of recombinant NoV VLPs covering a broad range of genotypes within both genogroups, the VHH tested were shown to be either genogroup specific, genotype specific, or strain specific. The different patterns of cross-reactivity were similar in nature to the observed behavior of conventional monoclonal antibodies [26,85–93]. The cross-reactivity observed by ELISA, together with the ability to function in neutralization surrogate assays utilizing different sources of carbohydrates (NoV VLP-HBGA, PGM and saliva blockade assays; and human RBC hemagglutination inhibition assays), all strongly suggest that some of the VHH may possess broad viral neutralizing activity, and that the nanobodies may prove successful as a prophylactic or therapeutic intervention.

The clones directed toward GI.1 NoV did not display cross reactivity to the other GI genotypes tested (GI.5 and GI.6) with the exception of the VHH N2, which also recognized GI.3 Desert Shield strain by ELISA. Within the GI specific VHHs, the clones N1, N5 and N9 all showed the best blockade performance within the different carbohydrate sources. The calculated EC_50_ values for these VHHs ranged between 0.275 and 2.0 μg/ml, all comparable to the EC_50_ values of the scFv derived from immunized chimpanzees (from 0.3 to 1.5 μg/ml) [41]. As therapy for the infection and diarrhea associated with GI.1 NoV, one strategy is a combination of the VHH antibodies N1 and N5, as they both illustrated optimal affinities similar to that reported for other VHHs directed to viral pathogens [94], the best overall blockade activity and they are directed toward different epitopes. This combination approach may diminish the selection of neutralization resistant variants during treatment [95,96].

The GII.4 genotype is further classified into variants that periodically shift to emerge as new pandemic strains [17]. Several of the VHH antibodies specific for GII.4 NoV not only showed cross-reactivity among the different variants of GII.4 tested from 1977 to 2012, but also showed cross-reactivity among other genotypes within the GII genogroup [13,22,23,79]. The GII.4 VHHs clones M3, M4 and M5 all showed similar CDR3 amino acid sequences. These data, together with the results from the competition assays, suggest these antibodies are directed toward the same epitope. Although they share the same CDR3, the VHH M3 displayed less cross-reactivity with other GII genotypes compared to VHH M5. This difference in specificity may ultimately be due to distinct amino acid changes in other regions of the sequence.

The VHH M1 and M5 showed optimal affinities to GII NoV VLPs, within the low nanomolar range, similar to those recently reported for other VHH directed to GII.4 and GII.10 NoV [80]. The VHH M1, and M5 showed the overall best blockade profile, with the EC_50_ values ranging between 0.341 and 2.0 μg/ml. Previously reported conventional MAbs with blockade capacity against GII.4 showed EC_50_ values from 0.119 to 0.737 μg/ml [13]. According to these EC_50_ values, comparable amounts of VHHs or conventional MAbs are needed to reach a similar blockade result [13]. Taking into account the size of the molecule, the comparison among the VHHs EC_50_ values indicates that around 20 times more molecules of VHH will be needed compared to the conventional MAbs. However, when comparing VHH to conventional MAbs for the design of an oral or parenteral therapy, the VHHs are advantageous compared to conventional MAbs because they do not need humanization and exhibit stability in the enteric tract environment [97]. Additionally, VHH show high expression yields in several biotechnological platforms: transgenic plants, yeast, baculovirus system, bacteria and mammal cells [43,45,47–49,98], that correlate with lower production costs [71]. The absence of an Fc domain and the binding of VHH to VLPs even in the presence of highly concentrated polyclonal antibodies suggest that an immune response following natural infection or vaccination would not interfere with VHH binding and activity in a therapeutic application.

The inconsistencies obtained in the results amongst the different blocking assays and HAI might be related to the fact that each source of carbohydrate represents different HBGAs surrounded by a different molecular environment. Therefore, in each particular case, different binding molecules might be involved. Furthermore, in the case of PGM, it was a complex mixture of carbohydrates wherein more than one type of HBGA was present (A, Ley and H2). Furthermore, HAI may be due not only to HBGA blocking, but also may be due to any other alteration in the VLP structure such us aggregation or disassembly of the VLPs. Additional work will be needed to define which surrogate neutralization screening assay or assays are optimal for prediction of protective efficacy.

Two GII specific VHH nanobodies were mapped in the VP1. The VHH M6 was the only one directed to a linear epitope as demonstrated by WB analysis. It proved to be broadly reactive with all the VLPs of the GII genotype tested. The linear epitope of VHH M6 was shown to be located at the C terminus of NoV VP1, within the P1 subdomain, in concordance with previous reports for cross reactive MAbs [89,92]. The epitope located within the protruding domain may be more accessible in the native virion than epitopes located in the S domain, the target of most other cross reactive MAbs [89,92] and VHH [80]. This VHH showed very high affinity in the picomolar range, with a k_d_ (k_off_) of 2.5 x10^-5^ s^-1^ indicative of a very slow dissociation, possibly due to the potential hydrophobic characteristic of the interaction. Despite the lack of carbohydrate blocking activity, this VHH may prove useful as an inhibitor of norovirus infection through a different mechanism, for example NoV particle disassembly as was reported for another VHH directed to a similar region in the P domain [80]. Additionally the very high affinity of this binder makes it suitable for NoV diagnosis. The GII.4 VHH M7, highly specific for MD2004 strain, was mapped to a conformational epitope in the P2 subdomain involving amino acids 340 and 376. This epitope had been predicted as the evolving epitope C [13] and this work constitutes the first confirmation of the predicted epitope with a monoclonal antibody.

The need for antiviral drugs in the control of norovirus diarrhea has been increasingly recognized [3]. The nanobodies described here, promise to be excellent reagents for use in NoV diagnostic assays and in basic research. Moreover, the known advantages of these small, safe, and stable molecules support their further evaluation as an attractive therapeutic option in the treatment of NoV gastroenteritis.

## Supporting Information

S1 Fig Western Blot analysis of the VHHs. a)VHH directed to GI.1 VP1, b)VHH directed to GII.4 VP1 and c) VHH M6 assayed by WB against VLPs of different genotypes.
(TIFF)
