# Supplementary figures and images for: Llama Nanoantibodies with Therapeutic Potential against Human Norovirus Diarrhea
Source: PLoS One. 2015 Aug 12;10(8):e0133665. doi: 10.1371/journal.pone.0133665 (PMC4534396; doi:10.1371/journal.pone.0133665)

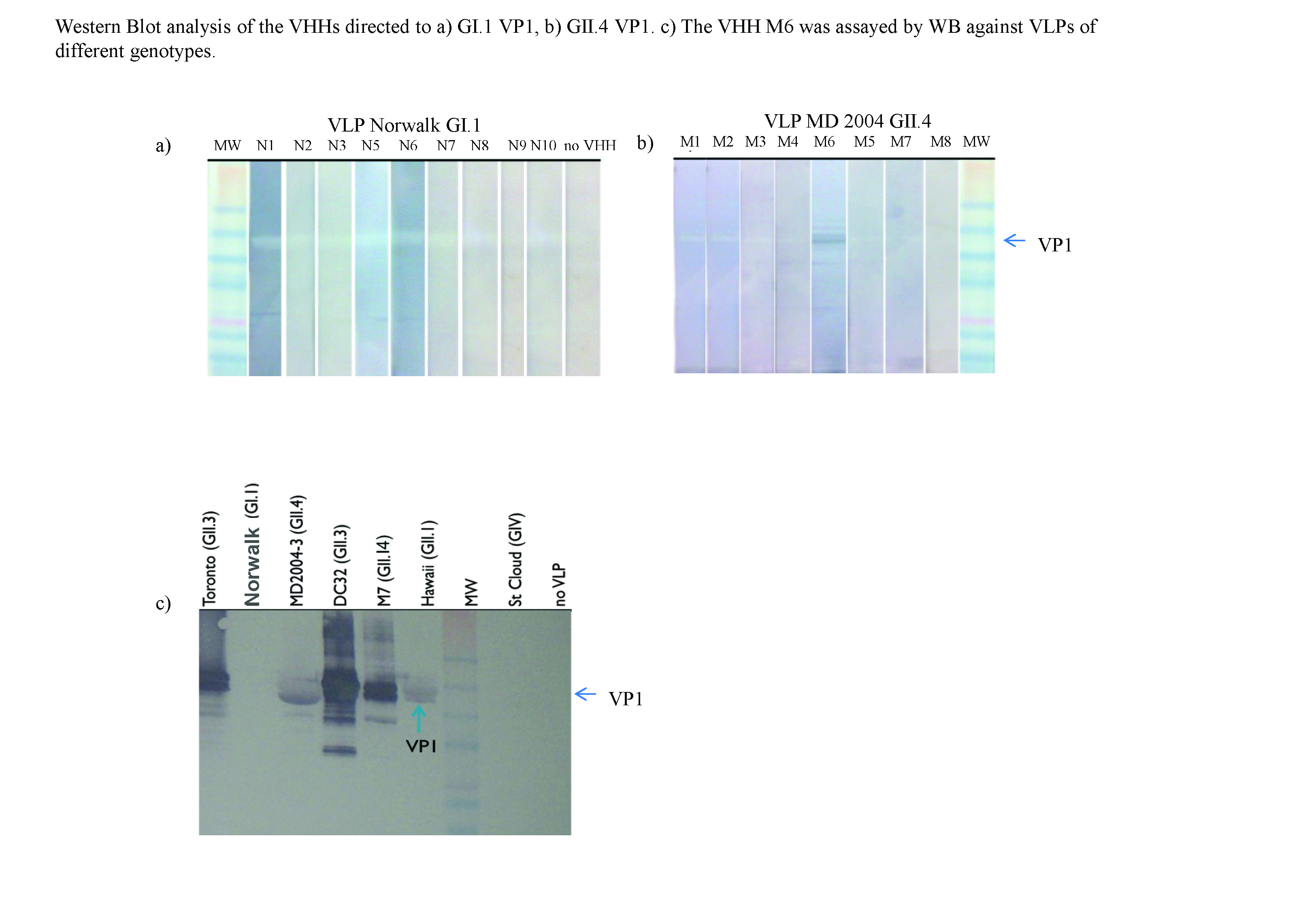

Supplement: S1 Fig — a)VHH directed to GI.1 VP1, b)VHH directed to GII.4 VP1 and c) VHH M6 assayed by WB against VLPs of different genotypes. (TIFF) [file pone.0133665.s001.tiff]
